# Service Robots as Work Support for Health Personnel in Long-Term Care: Protocol for a Scoping Review

**DOI:** 10.2196/89435

**Published:** 2026-07-08

**Authors:** Diego Losada-Floriano, Elin Thygesen, Filippo Sanfilippo, Michael Rygaard Hansen, Mariann Fossum

**Affiliations:** 1 Department of Health and Nursing Science Faculty of Health and Sports Sciences University of Agder Kristiansand, Agder Norway; 2 Department of Engineering Sciences Faculty of Engineering and Science University of Agder Kristiansand, Agder Norway

**Keywords:** health personnel, implementation science, long-term care, robotics, service robot

## Abstract

**Background:**

Demographic shifts are increasing the global demand for long-term care services, coinciding with a worldwide shortage of health care personnel. Service robots, designed to perform tasks in both professional and personal use, are perceived as a potential solution to alleviate health care personnel’s workload and enhance the quality of care. However, the existing literature is fragmented and heterogeneous, with a limited emphasis on the role of service robots in supporting residents rather than health care personnel. Furthermore, there is a lack of consistent definitions of service robotic technologies and a scarcity of studies on implementation models and frameworks.

**Objective:**

This scoping review aims to map and synthesize evidence regarding the implementation of service robots as work support for health care personnel in long-term care settings.

**Methods:**

A comprehensive 3-step search will be conducted in Embase, MEDLINE, APA PsycInfo, CENTRAL, Scopus, and CINAHL, along with gray literature databases and institutional repositories. Eligible sources encompass empirical studies and gray literature involving service robots, health care personnel, residents aged 65 years or older, and stakeholders such as informal caregivers within institutional long-term care. Exclusions apply to studies on home care, medical or industrial robots, and nonrobotic technologies. Data will be extracted and analyzed using the Joanna Briggs Institute methodology, with findings presented in tables, diagrams, and narrative summaries to identify gaps and inform future research and implementation strategies.

**Results:**

The project has been funded for a 4-year period starting in April 2025. This protocol was developed in October 2025 and subsequently registered in November 2025. A comprehensive search strategy was formulated and completely conducted on October 24, 2025. The screening of 4884 titles and abstracts was completed in December 2025, resulting in the retrieval of 64 (1.3%) full-text articles for eligibility assessment. Subsequent phases, including data extraction, analysis, evidence synthesis, and presentation of results, will be conducted sequentially. The scoping review is expected to be finalized by June 2026.

**Conclusions:**

This scoping review is expected to delineate the extent and characteristics of the existing evidence on service robots as work support for health personnel in long-term care settings. It will highlight the key reported outcomes and challenges encountered in implementation studies, as well as the theoretical frameworks, models, and concepts applied to address these issues.

**Trial Registration:**

Open Science Framework QWK58; https://osf.io/qwk58/

**International Registered Report Identifier (IRRID):**

PRR1-10.2196/89435

## Introduction

### Background

All nations are experiencing a profound demographic transition primarily driven by population aging. By the mid-2030s, the number of individuals aged 80 years or older is projected to exceed the number of infants under 1 year of age. Furthermore, by the late 2070s, the global population aged 65 years and above is expected to reach approximately 2.2 billion, surpassing those under 18 years [1]. These demographic shifts, both in size and proportion of the population of older adults, place considerable strain on health care systems by increasing the demand for long-term care services and intensifying the need for health care personnel and caregivers [2]. However, this growing demand is not matched by a proportional increase in the health care workforce [3], resulting in heavier workloads and a heightened risk of health care personnel burnout [2]. Although incremental progress is evident, with most countries for which data are available showing advancements in health workforce development and availability, the World Health Organization projects substantial global shortages of health care personnel by 2030, underscoring the urgent need for strategies that can expand workforce capacity and improve working conditions [3,4].

Within this context, the integration of robotics into health care, particularly service robots, has emerged as a promising and multidisciplinary domain within digital transformation initiatives in health and social care [5-7]. According to the International Organization for Standardization standard 8373:2021, a service robot is defined as a nonindustrial robot designed to perform useful tasks for individuals or equipment in personal or professional settings, such as handling or delivering items, transportation, cleaning and disinfection, inspection or surveillance, guidance or information, and physical assistance. This definition explicitly excludes medical and industrial robots and robotic devices, in line with the International Organization for Standardization standard 8373:2021 definition of service robot, which does not apply to these categories [8,9]. Given these applications, service robots are increasingly recognized as a technology with broad potential to support health care personnel by reducing physical workload, improving efficiency, and enhancing care quality [6,10]. For the purposes of this review, service robots as work support for health care personnel refer to professional or personal care service robots primarily used to support health personnel in task performance rather than patient-directed or indirect services, with informal caregivers considered stakeholders. Health care personnel include registered physicians and nurses, nursing assistants, allied health professionals, and other health care personnel directly involved in providing long-term care. Long-term care settings encompass nursing homes, assisted living facilities, residential care institutions, or equivalent housing care specially adapted for 24-hour services that provide continuous health and support services to older or dependent adults [11].

The volume of research on robots in health care has grown substantially over the past 5 years [5]. Within this body of evidence, a growing but heterogeneous set of studies indicates that service robots are being trialed or implemented in long-term care settings, where they can influence health care personnel’s workflows and experiences [6]. A preliminary search of Scopus, MEDLINE, and Embase via Ovid, PROSPERO, and Epistemonikos was conducted on September 9, 2025, and no systematic or scoping reviews focusing on the implementation aspects and models of service robots as work support in long-term care settings were identified. Existing scoping reviews primarily address the different types of robot-assisted care and their applications [12,13], health workers’ perceptions [14,15], and barriers to and facilitators of their implementation [16,17]. Additional evidence syntheses include an umbrella review summarizing robotics in nursing [6], 2 integrative reviews on health care robotics for older adults [18], and implementation challenges in nursing [19], as well as several systematic reviews examining service robot development methodologies [20]; health workers’ attitudes, perceptions, and preferences [2,21-23]; the impact of care robots on older adult care [24]; and the effectiveness of socially assistive robots (SARs) in nursing [10,25].

Nevertheless, significant conceptual and empirical gaps have persisted. First, existing syntheses disproportionately focus on SARs for residents’ well-being, making it difficult to isolate evidence on service robots explicitly deployed to support health care personnel in long-term care institutions. Second, knowledge regarding the effectiveness, cost-efficiency, and implementation models of service robots in real-world long-term care environments is limited [2,6,13,22,24]. Evidence on sustainable business models for municipal care is minimal, and robust economic evaluations remain scarce [21,24]. Finally, current reviews exhibit inconsistent definitions and classifications of health care robots, and little is known about the impact of service robots on workflows, task allocation, required competencies, or job satisfaction among health care personnel [2,10,24,25].

To address these gaps and clarify key concepts, this scoping review aims to identify and synthesize the available evidence on the adoption and integration of service robots to support health care personnel by assuming routine tasks for older adults in long-term care settings. By mapping existing research, this review provides a foundation for future empirical studies on implementation strategies for service robots in health care. It will also identify areas and processes where implementation is needed to improve working conditions and enhance care quality while highlighting opportunities for designing robotic systems that promote both efficiency and well-being in municipal health and care services.

### Review Question

The review question is as follows: what evidence exists on the adoption and integration of service robots in long-term care settings, specifically regarding their role in supporting health care personnel and informal caregivers in performing routine tasks?

The review subquestions are as follows:

- What are the key concepts and definitions associated with robotics in health care, with a particular focus on service robots?
- What methodologies, target populations, and reported outcomes are described in the implementation studies of service robots used to support health care personnel in performing routine tasks?
- What types of evidence, theoretical frameworks, or conceptual models have been used to examine the implementation of service robots in long-term care settings to support health care personnel, older adult residents, and caregivers?

## Methods

### Study Design

This scoping review will follow the Joanna Briggs Institute (JBI) methodology [26]. A PRISMA-ScR (Preferred Reporting Items for Systematic Reviews and Meta-Analyses extension for Scoping Reviews) checklist was used for reporting and can be found in Multimedia Appendix 1 [27]. The objectives, inclusion criteria, and methods are documented in this protocol, which is registered in the Open Science Framework. The inclusion and exclusion criteria are presented in Table 1.

**Table 1:** Inclusion and exclusion criteria for the identified studies.

Key concept	Inclusion criteria	Exclusion criteria
Population	Health personnel; studies including health personnel and individuals aged ≥65 y residing in long-term care or their relatives or informal caregivers as stakeholders	Studies focusing solely on older adults living at home, home health care, in-home nursing, or family caregiving unrelated to institutional long-term care
Concept	Service robots, defined as robots that perform useful tasks for humans or equipment for personal or professional uses	Industrial robots or industrial automation applications, medical robots, training or education robots, robotic devices, exoskeletons, socially assistive robots used only for entertainment or therapy in home care, and nonrobotic welfare technologies
Context	Long-term care settings (nursing homes, assisted living facilities, and residential care institutions) and mixed contexts with distinct long-term care data	Home health care for older adults, in-home care, private residences, specialist health care, and acute hospitals; laboratory-only studies without care context
Types of evidence sources and study designs	Peer-reviewed primary studies; evidence syntheses; tertiary evidence; and gray literature from verifiable sources focusing on theses and dissertations with institutional records, policy reports, technical guidelines, and institutional documents	Unverified, unpublished, ambiguous, or nonauthenticated literature without traceable provenance
Publication types	All other publication types	Letters, editorials, conference abstracts, and study protocols
Language	Studies and gray literature in English, Spanish, Italian, German, or Scandinavian languages in which there is author proficiency; other languages if translation is feasible	Studies in languages for which translation via University of Agder academic services or the DeepL translator is not feasible

### Eligibility Criteria

#### Participants

This review will include empirical studies and gray literature involving health care personnel such as registered physicians and nurses, nursing assistants, allied health professionals, and other personnel directly engaged in providing long-term care. Studies including mixed populations, health personnel, and individuals aged 65 years or older, as well as informal caregivers or relatives, will also be considered for inclusion.

Specific exclusion criteria are studies focusing exclusively on older adults living at home, home health care services, in-home nursing care, or family caregiving without relevance to institutional long-term care facilities.

#### Concept

The core concept of this scoping review is service robots, defined as robots for personal or professional use that perform useful tasks for humans or equipment. In personal use, tasks may include handling or serving items, transportation, physical support, guidance or information, grooming, cooking, handling food, and cleaning. In professional use, tasks may involve inspection, surveillance, item handling, personal transportation, guidance or information, cooking, handling food, and cleaning [8,9].

Specific exclusion criteria are studies addressing industrial robots or industrial automation applications, medical robots (eg, surgical or therapeutic and rehabilitation robots), training or education robots, robotic devices, exoskeletons, SARs used solely for entertainment or therapy in home care, and nonrobotic welfare technologies.

#### Context

This review will consider studies examining the adoption and integration of service robots as work support in long-term care settings. These settings include nursing homes, assisted living facilities, residential care institutions, or equivalent housing care specially adapted for 24-hour services that provide continuous health and support services to older or dependent adults [11].

Specific exclusion criteria are studies focusing on home health care for older adults, in-home care settings, private residences, specialist health care, or acute hospitals and laboratory-only studies without a care context.

#### Types of Sources

This scoping review will encompass experimental and quasi-experimental designs, including randomized controlled trials, non–randomized controlled trials, before-and-after studies, and interrupted time-series studies. Additionally, it will consider mixed methods or multi-methods designs and analytical observational studies, such as prospective and retrospective cohort studies, case-control studies, and analytical cross-sectional studies, as well as descriptive observational designs, specifically descriptive cross-sectional studies.

Qualitative studies using methodologies such as phenomenology, grounded theory, ethnography, qualitative descriptions, action research, and feminist research will also be included. Systematic reviews that satisfy the inclusion criteria will be considered.

To ensure the breadth and comprehensiveness of the search strategy [26], this review incorporates gray literature with a focus on verifiable sources such as published theses, policy reports, technical guidelines, and institutional documents relevant to the research question.

Specific exclusion criteria for types of sources are literature derived from sources that are unverifiable, inaccessible, or unconfirmed, as well as unpublished or ambiguous documents lacking known provenance. Letters, editorials, conference abstracts, and study protocols will also be excluded.

### Search Strategy

The search strategy aims to identify both published and unpublished studies using 3 steps. First, an initial limited search of Scopus, MEDLINE, and Embase via Ovid, PROSPERO, and Epistemonikos will be conducted using selected keywords to identify relevant studies. The text words in the titles and abstracts of retrieved articles, as well as index terms, will then be analyzed to develop a comprehensive search strategy. Second, a full search strategy using all identified keywords and index terms will be developed and adapted for each included database and information source. Finally, the reference lists of all included articles and reports will be screened for additional studies. Complementary searches will be performed using specialized artificial intelligence research assistant tools: Ai2 Asta (current version 2025), developed by the Allen Institute for Artificial Intelligence, and Scopus AI (current version 2025), accessible via the University of Agder library’s Scopus account and developed by Elsevier. Multimedia Appendix 2 provides the comprehensive search strategy, and Multimedia Appendix 3 details the prompts and records obtained from the artificial intelligence research assistant tools, ensuring transparency and reproducibility.

The databases to be searched are Embase, MEDLINE, CENTRAL, and APA PsycInfo via Ovid; Scopus; CINAHL (EBSCO); and ProQuest Dissertations and Theses Global via Web of Science. At the University of Agder library, articles from the full-text databases IEEE Xplore and ACM Digital Library are indexed in Scopus, thereby ensuring their inclusion in our search strategy. There are no restrictions on the inclusion of sources based on language or year of publication. However, studies published in languages for which translation is not feasible using the academic translation services available to the staff at the University of Agder or the DeepL translator will be excluded.

The search for gray literature will concentrate on standards, guidelines, policies, and regulations regarding the use of robots in health care [28] and will include Google Scholar, ProQuest Dissertations and Theses Global, OpenGrey, Data Archiving and Networked Services, APA PsycExtra, the Social Science Research Network, institutional repositories (the World Health Organization, United Nations, European Parliament and European Commission, DiVA portal, Evidence for Policy and Practice Information Centre, National Institute for Health and Care Excellence, and Trip medical database), and governmental websites (the Social Care Institute for Excellence; National Health Service in England; UK government; Japan Ministry of Health, Labor, and Welfare; Agency for Healthcare Research and Quality; National Information Center on Health Services Research and Health Care Technology; Public Health Agency of Canada; and Norwegian Institute of Public Health).

### Study and Source of Evidence Selection

**Figure 1:**
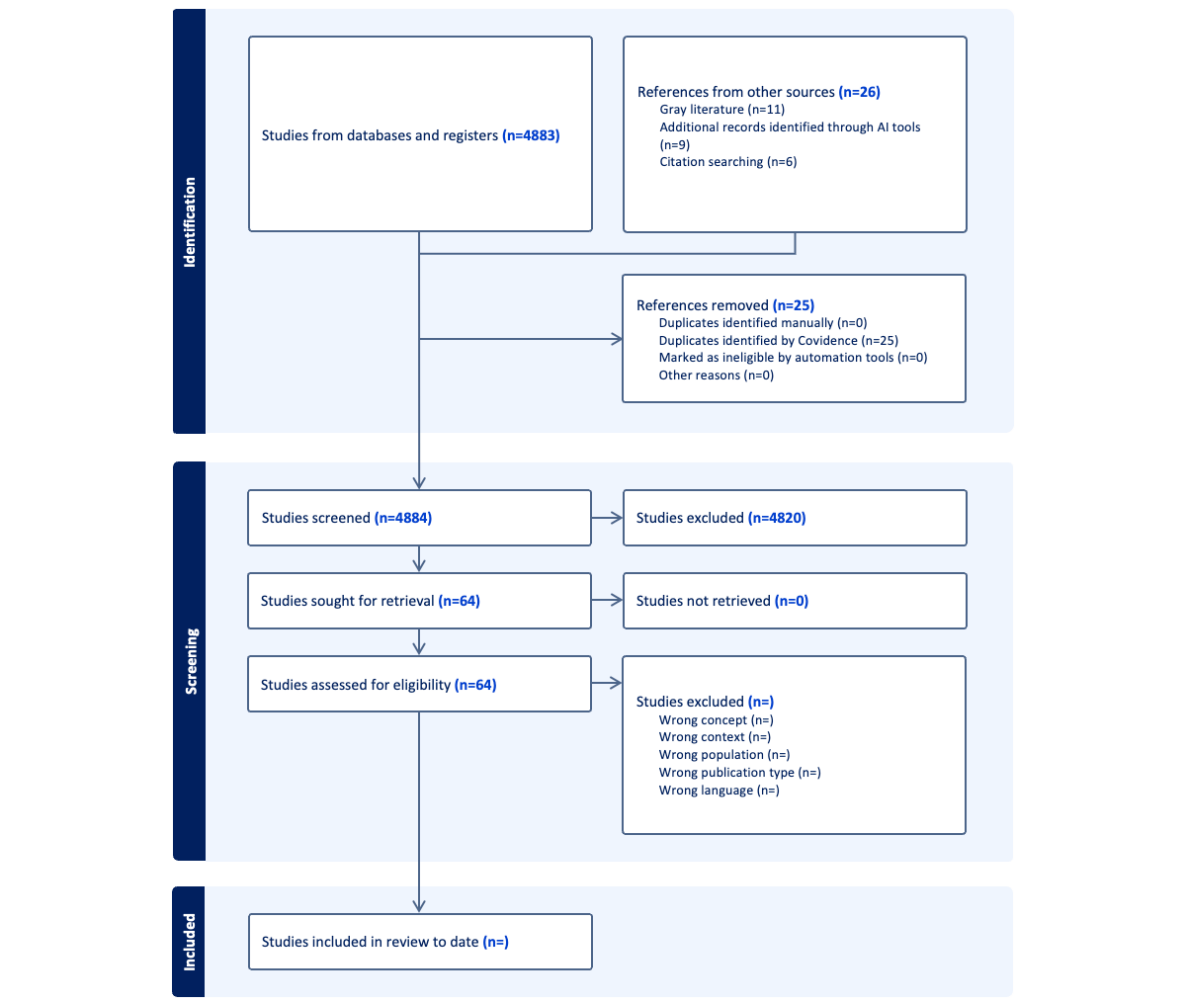
PRISMA (Preferred Reporting Items for Systematic Reviews and Meta-Analyses) flowchart. AI: artificial intelligence.

All identified citations will be imported into EndNote 2025.1 (Clarivate Analytics) and Covidence (Veritas Health Innovation), and duplicates will be removed. After a pilot test, titles and abstracts will be independently screened by 2 reviewers (DL-F and MF) against the inclusion criteria. Full texts of potentially relevant sources will be retrieved and assessed in the JBI SUMARI software [29]. The full texts of the selected citations will be assessed in detail against the inclusion criteria by 2 independent reviewers (DL-F and MF). Reasons for exclusion at the full-text review stage will be recorded and reported. Any disagreements that arise between the reviewers at each stage of the selection process will be resolved through discussion or by consulting a third reviewer (ET). The results of the search and the study inclusion process will be reported in full in the final scoping review and presented in a PRISMA (Preferred Reporting Items for Systematic Reviews and Meta-Analyses) flow diagram (Figure 1) [27].

### Data Extraction

Two reviewers (DL-F and MF) will independently extract data from 3 full-text sources as a pilot extraction to ensure that all relevant information is included. Subsequently, extraction of all data will be conducted independently by 2 reviewers (DL-F and MF) using a data extraction tool developed by the reviewers and adapted from the JBI template for source of evidence details, characteristics, and results extraction instrument [26]. The extracted data will include details on participants, concepts, contexts, study methods, and key findings relevant to the review objective. A draft chart is provided in Multimedia Appendix 4. The draft data extraction tool will be modified and refined as needed during the extraction, and the modifications will be documented. The authors of the papers will be contacted to request missing or additional data where appropriate.

### Data Analysis and Presentation

We will adopt a collaborative, team-based approach for the analysis and presentation of the results [30]. DL-F will conduct the preliminary analysis, following which the team will convene regularly, either through in-person meetings or via email throughout the review process. Contributions from MF, ET, FS, and MRH will be incorporated into the final draft through consensus.

The extracted data will be presented in tables and diagrams, aligned with the aims and goals of this scoping review. Accompanying the tabular or charted data will be a narrative summary that outlines the characteristics of the literature on service robots as work support in long-term care settings, explaining how the findings connect to the review’s objective and question and highlighting any gaps in the existing literature. The data presentation will include categories such as publication year, country, purpose, service robot definition, applications, design, population, intervention details, models, frameworks for implementation, and key findings. The categories may be adjusted based on these findings. If more than 80 studies are included, an evidence and gap map will be provided to visually represent the findings [31].

## Results

The project has been funded for a 4-year period starting in April 2025. This scoping review protocol was developed in October 2025 and registered with the Open Science Framework on November 7, 2025. A comprehensive search strategy designed and executed by a University of Agder librarian was applied on October 24, 2025. The search covered multiple databases: Embase, MEDLINE, CENTRAL, and APA PsycInfo via Ovid; Scopus; CINAHL (EBSCO); and ProQuest Dissertations and Theses Global via Web of Science.

As described in Figure 1, the combined search identified 4883 records, with an additional 26 records retrieved from other sources. Of these 4909 records, after removing 25 (0.5%) duplicates using Covidence, 4884 (99.5%) unique records were uploaded for screening. During the title and abstract screening phase completed in December 2025, of the 4884 studies, 4820 (98.7%) were excluded, leaving 64 (1.3%) studies for full-text review for eligibility assessment. To date, 27 studies have been included. The results of the study inclusion and subsequent stages of this scoping review are anticipated to be completed by the end of June 2026, with submission for publication planned for August 2026.

## Discussion

This ongoing scoping review is anticipated to provide a comprehensive overview of the variety, characteristics, and applications of service robots supporting health personnel in long-term care settings. It seeks to inform the theories, models, and frameworks related to service robot implementation, as well as identifying key reported effects on health personnel’s workflow and workload alongside challenges and gaps in implementation. Additionally, the review will clarify essential concepts and classifications of service robots as work support for health personnel in long-term care, establishing a foundation for future empirical research on implementation strategies within the health care sector, with a particular focus on municipal health and care services.

Previous scoping reviews on robotics in health care, including service robots, have primarily concentrated on the types and uses of robot-assisted care in general [12,13], health care professionals’ perspectives [14,15], and implementation barriers and facilitators [16,17]. Moreover, the literature is currently dominated by studies on SARs aimed at enhancing the well-being of individuals in older adult care [12]. Therefore, a scoping review specifically focusing on service robots as work support for health personnel in long-term care is expected to provide novel and targeted evidence.

The proposed methodology has several strengths. First, by mapping the breadth of existing and emerging primary studies as well as secondary, tertiary, and gray literature focused on service robots supporting health personnel’s work, this review will offer a comprehensive and up-to-date synthesis of the research landscape. Second, by including all study methodologies, the review will provide valuable insights into the research approaches applied to service robots in long-term care. Finally, the flexibility and prespecified methodological features of the scoping review as a systematic evidence synthesis will facilitate an evaluation of service robots as an emerging and complex digital health intervention within municipal health and care services [26].

This scoping review also faces certain limitations. The heterogeneity of the included studies, stemming from diverse evidence types and data sources, may complicate comparisons and introduce inconsistencies in data analysis and synthesis. Moreover, the decision not to conduct a critical appraisal of included studies limits the ability to assess evidence quality. Finally, rapid advancements in robotics and digital health may quickly render the review findings outdated [26,32].

By synthesizing current research, this review aims to establish a foundation for future studies on implementing service robots as work support for health personnel in municipal health and care services. It will also identify critical areas where integrating service robots can improve working conditions for health personnel and enhance care quality. The dissemination plan includes registering the protocol in the Open Science Framework repository to ensure transparency and reproducibility; publishing both the protocol and the completed review in an open access, peer-reviewed journal; and sharing findings through conference presentations and stakeholder-targeted summaries. Where relevant, results will be communicated to the research team, institutional partners, and professional networks to support future implementation and research translation.

## Appendix

Multimedia Appendix 1 PRISMA-ScR (Preferred Reporting Items for Systematic Reviews and Meta-Analyses extension for Scoping Reviews) checklist.

Multimedia Appendix 2 Search strategy.

Multimedia Appendix 3 Additional references identified using artificial intelligence research assistant tools.

Multimedia Appendix 4 Data extraction instrument.

## Abbreviations

JBI: Joanna Briggs Institute
PRISMA: Preferred Reporting Items for Systematic Reviews and Meta-Analyses
PRISMA-ScR: Preferred Reporting Items for Systematic Reviews and Meta-Analyses extension for Scoping Reviews
SAR: socially assistive robot
