# Supplementary material for: Service Robots as Work Support for Health Personnel in Long-Term Care: Protocol for a Scoping Review
Source: JMIR Res Protoc. 2026 Jul 8;15:e89435. doi: 10.2196/89435 (PMC13392531; doi:10.2196/89435)
Supplement: Multimedia Appendix 2 [file resprot_v15i1e89435_app2.pdf]

Protocol

Service Robots as Work Support for Health Personnel in Long-term Care: Protocol for a Scoping Review

Diego Losada-Floriano MD, MSc; Elin Thygesen PhD; Filippo Sanfilippo PhD; Michael Rygaard Hansen PhD; Mariann Fossum PhD.

Multimedia Appendix 2. Search Strategy

Search concepts:

1. Concept: Service robots (Words from title only).
2. Context: Long term care (Words from title, abstracts, or key/index words).
3. Exclude: Surgical or intensive care contexts.
4. Exclude: Publication types, letter or editorials.

Database: Scopus. The search was conducted on October 24, 2025.

((TITLE(robot* w/5 (caregiver* or carer* or humanoid or caring or carrier* or Autonom* or ai OR "artificial intell*" or companion* or collaborat* or aid OR aided* OR ambient* or healthcare or aged or old or older or elder* or aging or nursing or "long term" or residents* or interact* or service* or support* or social* or assist* or mobil* or transport* or laundry or personal* or domestic* or care or cleaning or clean or disinfection* or inspection* or maintenance or logistic* or hospitality or information* or telepresence* or handling or serving or cooking)) OR TITLE(carebot* OR "autonomous agent*" OR "telepresence device*" OR "telepresence system*" OR "social agent*" OR "embodied agent*" OR "embodied technolog*")) AND TITLE-ABS-KEY("nursing home*" or institution* or residential* or facilities or facility or "long term care" or "care home*" or "group home*" or "Halfway House*" or "Home* for the Aged" or residents* or "elder* care center*" or healthcare or "health care" or "nursing care")) AND NOT (TITLE-ABS-KEY(surgery OR surgical* OR "intensive care" OR "critical* care" OR laparoscop* OR acute OR emergency OR perioperativ* OR intraoperativ* OR peri-operativ* OR intra-operativ* OR postoperativ* OR post-operativ* OR Endoscop* OR diagnos*) or DOCTYPE(le OR ed))

Results: n = 4270

Database: Ovid databases simultaneously searched. EBM Reviews - Cochrane Central Register of Controlled Trials <September 2025>; Embase <1974 to 2025 Week 42>;Ovid MEDLINE(R) ALL <1946 to October 23, 2025>;APA PsycInfo <1806 to October 2025 Week 3>. The search was conducted on October 24, 2025.

#	Query	Records retrieved
1	(robot* adj6 (caregiver* or carer* or humanoid or caring or carrier* or Autonom* or ai or "artificial intell*" or companion* or collaborat* or aid or aided* or ambient* or healthcare or aged or old or older or elder* or aging or nursing or "long term" or residents* or interact* or service* or support* or social* or assist* or mobil* or transport* or laundry or personal* or domestic* or care or	66682

	cleaning or clean or disinfection* or inspection* or maintenance or logistic* or hospitality or information* or telepresence* or handling or serving or cooking)).ti.	
2	(carebot* or "autonomous agent*" or "telepresence device*" or "telepresence system*" or "social agent*" or "embodied agent*" or "embodied technolog*").ti.	403
3	1 or 2	67066
4	("nursing home*" or institution* or residential* or facilities or facility or "long term care" or "care home*" or "group home*" or "Halfway House*" or "Home* for the Aged" or residents* or "elder* care center*" or healthcare or "health care" or "nursing care").ti,ab,hw,kf,id,kw.	6727010
5	3 and 4	11103
6	(letter* or editorial*).pt. or (surgery or surgical*).mp. or exp Specialties, Surgical/ or exp Surgical Procedures, Operative/ or ("intensive care" or "critical* care" or laparoscop* or acute or emergency or perioperativ* or intraoperativ* or peri-operativ* or intra-operativ* or postoperativ* or post-operativ* or Endoscop* or diagnos*).ti,ab,kf,kw,id,hw.	29736974
7	5 not 6	1956
8	remove duplicates from 7	1312

Results:

7	5 not 6<br><br>EBM Reviews - Cochrane Central Register of Controlled Trials <September 2025><br><br>Embase <1974 to 2025 Week 42><br><br>Ovid MEDLINE(R) ALL <1946 to October 23, 2025><br><br>APA PsycInfo <1806 to October 2025 Week 3>	1956<br><br>98<br><br>763<br><br>733<br><br>362

Database: CINAHL (EBSCO). Notice: "The default fields for unqualified searches consist of the following: Title, Abstract and Subject headings, PubMed ID (PMID), Digital Object Identifier, Author" (EBSCO. CINAHL - Database Help)[1]. The search was conducted on October 24, 2025.

((TI (robot* N5 (caregiver* or carer* or humanoid or caring or carrier* or Autonom* or ai OR "artificial intell*" or companion* or collaborat* or aid OR aided* OR ambient* or healthcare or aged or old or older or elder* or aging or nursing or "long term" or residents* or interact* or

[1] MH = Exact CINAHl Headings, + expand with narrowing terms. TI = Words from title

service* or support* or social* or assist* or mobil* or transport* or laundry or personal* or domestic* or care or cleaning or clean or disinfection* or inspection* or maintenance or logistic* or hospitality or information* or telepresence* or handling or serving or cooking)) OR TI (carebot* OR "autonomous agent*" OR "telepresence device*" OR "telepresence system*" OR "social agent*" OR "embodied agent*" OR "embodied technolog*")) AND ("nursing home*" or institution* or residential* or facilities or facility or "long term care" or "care home*" or "group home*" or "Halfway House*" or "Home* for the Aged" or residents* or "elder* care center*" or healthcare or "health care" or "nursing care")) NOT (surgery OR surgical* OR MH "Specialties, Surgical+" OR MH "Surgery, Operative+" OR "intensive care" OR "critical* care" OR laparoscop* OR acute OR emergency OR perioperativ* OR intraoperativ* OR peri-operativ* OR intra-operativ* OR postoperativ* OR post-operativ* OR Endoscop* OR diagnos*)

Filter: EXCLUDE MEDLINE

Search mode: Find all my search terms

Results: n = 372

Database: ProQuest Dissertations and Theses Global (through Web of Science). The search was conducted on October 24, 2025.

((TI=(robot* NEAR/5 (caregiver* or carer* or humanoid or caring or carrier* or Autonom* or ai OR "artificial intell*" or companion* or collaborat* or aid OR aided* OR ambient* or healthcare or aged or old or older or elder* or aging or nursing or "long term" or residents* or interact* or service* or support* or social* or assist* or mobil* or transport* or laundry or personal* or domestic* or care or cleaning or clean or disinfection* or inspection* or maintenance or logistic* or hospitality or information* or telepresence* or handling or serving or cooking)) OR TI=(carebot* OR "autonomous agent*" OR "telepresence device*" OR "telepresence system*" OR "social agent*" OR "embodied agent*" OR "embodied technolog*")) AND TS=("nursing home*" or institution* or residential* or facilities or facility or "long term care" or "care home*" or "group home*" or "Halfway House*" or "Home* for the Aged" or residents* or "elder* care center*" or healthcare or "health care" or "nursing care")) NOT TS=(surgery OR surgical* OR "intensive care" OR "critical* care" OR laparoscop* OR acute OR emergency OR perioperativ* OR intraoperativ* OR peri-operativ* OR intra-operativ* OR postoperativ* OR post-operativ* OR Endoscop* OR diagnos*)

Results: 183
