# Supplementary material for: Service Robots as Work Support for Health Personnel in Long-Term Care: Protocol for a Scoping Review
Source: JMIR Res Protoc. 2026 Jul 8;15:e89435. doi: 10.2196/89435 (PMC13392531; doi:10.2196/89435)
Supplement: Multimedia Appendix 3 [file resprot_v15i1e89435_app3.pdf]

Protocol

Service Robots as Work Support for Health Personnel in Long-term Care: Protocol for a Scoping Review

Diego Losada-Floriano MD, MSc; Elin Thygesen PhD; Filippo Sanfilippo PhD; Michael Rygaard Hansen PhD; Mariann Fossum PhD.

Multimedia Appendix 4. Additional references identified through AI-tools

Source: Ai2 Asta. Version available at https://asta.allen.ai, developed by the Allen Institute for Artificial Intelligence (2025). The search was conducted on December 16, 2025.

#	Prompt	Records retrieved
**1**	*"I need to identify the available evidence on the adoption and integration of service robots to support healthcare personnel by assuming routine tasks for older adults in long-term care settings"*	20 Perfectly Relevant; 66 Relevant; 172 Somewhat Relevant

References retrieved:

1. Abbott R, Noreen O, McGill P, Whear R, Bethel A, Garside R, et al. How do "robopets" impact the health and well‐being of residents in care homes? A systematic review of qualitative and quantitative evidence. International Journal of Older People Nursing. 2019;14.
2. Abraham M, Dominic AA, Mathew J, McLane P, Cummings G, Holroyd-Leduc J. Impact of community paramedic interventions on transfers from long term care to emergency departments: results of a systematic review. Canadian Journal of Emergency Medicine. 2021;24:101 – 2.
3. Adam P, Fosch-Villaronga E, Burmeister O. Cybersecurity, value sensing robots for LGBTIQ+ elderly, and the need for revised codes of conduct. Australas J Inf Syst. 2020;24.
4. Adeyemo A, Alice C, Liz K. Utilisation of robots in nursing practice: an umbrella review. BMC Nursing. 2025;24.
5. Adi B, Ela L-P, Oron-Gilad T. Bridging the gap: Generating a design space model of Socially Assistive Robots (SARs) for Older Adults using Participatory Design (PD). 2022.
6. Albarqi M. Exploring the Effectiveness of Technology-Assisted Interventions for Promoting Independence in Elderly Patients: A Systematic Review. Healthcare. 2024;12.
7. Alexandra T, Andreas U, Hartmut S, Manser T. Older Adults' Engagement and Mood During Robot-Assisted Group Activities in Nursing Homes: Development and Observational Pilot Study. JMIR rehabilitation and assistive technologies. 2023;10:e48031.
8. Anastasia KO, Christina NH, Breazeal C, Hae Won P. Personal Narratives in Technology Design: The Value of Sharing Older Adults' Stories in the Design of Social Robots. Frontiers in Robotics and AI. 2021;8.
9. Anastasia KO, Jennifer Z, Cynthia B, Hae Won P. Promising directions for human-robot interactions defined by older adults. Frontiers in Robotics and AI. 2024;11.
10. Andrea Antonio C, Mariarosaria E, Perillo F, Marco R, Sebillo M, Vitiello G. Enhancing Elderly Health Monitoring: Achieving Autonomous and Secure Living through the Integration of Artificial Intelligence, Autonomous Robots, and Sensors. Electronics. 2023.
11. Andrew H, Julia S, Severin K. Towards Equitable Agile Research and Development of AI and Robotics. ArXiv. 2024;abs/2402.08242.
12. Anghel I, Cioara T, Dorin M, Marcel A, Claudia P, Salomie I, et al. Smart Environments and Social Robots for Age-Friendly Integrated Care Services. International Journal of Environmental Research and Public Health. 2020;17.
13. Ankit AR, Seyed Amir T, Luces JVS, Fumi S, Hirata Y. CARE: Cooperation of ai Robot Enablers to Create a Vibrant Society. IEEE Robotics \& Automation Magazine. 2023;30:8–23.
14. Anna H, Guy L, Emily SC. What Makes a Robot Social? A Review of Social Robots from Science Fiction to a Home or Hospital Near You. Current Robotics Reports. 2021;2:9 – 19.
15. Antonio Miguel C, Hector Perez Lopez P, Daum C, Rutledge E, King S, Lili L. Technology Acceptance and Usability of a Mobile App to Support the Workflow of Health Care Aides Who Provide Services to Older Adults: Pilot Mixed Methods Study. JMIR Aging. 2022;5.
16. Archibald M, Barnard A. Futurism in nursing: Technology, robotics and the fundamentals of care. Journal of Clinical Nursing. 2018;27:2473–80.

17. Arne M, Bischof A, Jannis H, Benjamin L. A critique of robotics in health care. Ai \& Society. 2021;37:467 – 77.
18. Arto L, Marketta N, Jari P. Demands of Dignity in Robotic Care. Techn \'e : Research in Philosophy and Technology. 2019.
19. Badr N, Maha D. Assistive Healthcare Robotics - Challenges in Nursing Service Innovation: Critical Review. 2022.
20. Bahar I, Sanna K, Gabriel S. Recommendations for designing conversational companion robots with older adults through foundation models. Frontiers in Robotics and AI. 2024;11.
21. Baptiste I, Maribel P, Manon L, Samuel B, Rigaud A. Social Telepresence Robots: A Narrative Review of Experiments Involving Older Adults before and during the COVID-19 Pandemic. International Journal of Environmental Research and Public Health. 2021;18.
22. Bartosz S, Tobis S, Baum E, Suwalska A, Kropińska S, Katarzyna S, et al. Robots for Elderly Care: Review, Multi-Criteria Optimization Model and Qualitative Case Study. Healthcare. 2023;11.
23. Bedaf S, Marti P, Amirabdollahian F, Witte LDd. A multi-perspective evaluation of a service robot for seniors: the voice of different stakeholders. Disability and Rehabilitation: Assistive Technology. 2018;13:592 – 9.
24. Bogoslov I, Sorina C, Lungu A. Perspectives on Artificial Intelligence Adoption for European Union Elderly in the Context of Digital Skills Development. Sustainability. 2024.
25. Boyuan W, Shanji C, Gexin X. Advancing healthcare through mobile collaboration: a survey of intelligent nursing robots research. Frontiers in Public Health. 2024;12.
26. Broadbent E, Rebecca QS, MacDonald B. Acceptance of Healthcare Robots for the Older Population: Review and Future Directions. International Journal of Social Robotics. 2009;1:319–30.
27. Broadbent E, Rie T, Anna P, Knock B, Kerse N, Day K, et al. Attitudes towards health ‐ care robots in a retirement village. Australasian Journal on Ageing. 2012;31.
28. Broekens J, Marcel H, Rosendal H. Assistive social robots in elderly care: a review. Caries Research. 2009.
29. Buchanan C, Lyndsay H, Rita W, Booth R, Tracie LR, Bamford M, et al. Nursing in the Age of Artificial Intelligence: Protocol for a Scoping Review. JMIR Research Protocols. 2020;9.
30. Buchanan C, Lyndsay H, Rita W, Richard GB, Tracie LR, Bamford M. Predicted Influences of Artificial Intelligence on the Domains of Nursing: Scoping Review. JMIR Nursing. 2020;3.
31. Caren H, Eva Theresa J, Martin M. Integrating the Perspectives of Relevant Stakeholders into the Development of a Service Robot for Nursing Homes: Needs Analysis and Scenario Development Using the International Classification of Functioning, Disability and Health (ICF). International Journal of Social Robotics. 2025;17:1725 – 45.
32. Catrin M. A Softwaremodule for an Ethical Elder Care Robot. Design and Implementation Abstract:. Ethics in Progress. 2019.
33. Cavallo F, Bevilacqua R. Acceptance of Robot-Era system: results of robotic services in smart environments with older adults. 2018.
34. Cavallo F, Raffaele E, Raffaele L, Manzi A, Bevilacqua R, Elisa F, et al. Robotic Services Acceptance in Smart Environments With Older Adults: User Satisfaction and Acceptability Study. Journal of Medical Internet Research. 2018;20.
35. Celia Nieto A, Pfingsthorn M, Gliesche P, Eichelberg M, Hein A. A Survey of Robotic Systems for Nursing Care. Frontiers in Robotics and AI. 2022;9.
36. Chantal K, Birgit L. Designing for flourishing: a conceptual model for enhancing older adults' well-being with social robots. Frontiers in Robotics and AI. 2025;12.
37. Chapman S, Jacqueline RM, Spetz J. Emerging Health Technologies in Long-Term Care and Suppliers' Views on Their Potential to Assist and Support the Workforce. Medical Care Research and Review. 2023;80:619 – 30.
38. Che R, Ruan Y, Kodate N, Yiwen S, Xiaoting L, Donnelly S, et al. Effectiveness and usability of care robots in supporting older adults living with frailty: A systematic review. Digital Health. 2025;11.
39. Cheonshu P, Sangseung K, Jaehong K, Jinhwan O. A study on service robot system for elder care. 2012 9th International Conference on Ubiquitous Robots and Ambient Intelligence (URAI). 2012:546–7.
40. Chia-Rong L, Edward THC, Min-Jing S, Li-Tsai L, Mei-Zhen H, Ching-Chih H. Application of Indoor Positioning Systems in Nursing Homes: Enhancing Resident Safety and Staff Efficiency. Sensors (Basel, Switzerland). 2024;24.
41. Chongke W, Szep J, Hariri S, Nimit KA, Sumit KA, Nevarez C. SeVA: An AI Solution for Age Friendly Care of Hospitalized Older Adults. 2021.

42. Chrisantus E. Internet of Things Meets Robotics: A Survey of Cloud-based Robots. ArXiv. 2023;abs/2306.02586.
43. Christoforou E, Avgousti S, Ramdani N, Novales C, Panayides A. The Upcoming Role for Nursing and Assistive Robotics: Opportunities and Challenges Ahead. Frontiers in Digital Health. 2020;2.
44. Christoph O, Nicole S, Warmbein A, Rathgeber I, Fischer U, Inge E. Service Robotics in Nursing Care. The Preliminary Results of a Scoping Review. Studies in health technology and informatics. 2021;281:1075–6.
45. Christoph O, Nicole S, Warmbein A, Rathgeber I, Mehler-Klamt A, Fischer U, et al. Assistive robotic systems in nursing care: a scoping review. BMC Nursing. 2023;22.
46. Claire S, Michael T, John M, Maggie H, Ari BC, Andrew N, et al. Abstracts from the 2024 Annual Scientific Meeting of the Canadian Academy of Geriatric Psychiatry and Canadian Coalition for Seniors' Mental Health. Canadian Geriatrics Journal. 2025;28:183 – 211.
47. Consuelo G, Maribel P, Grgory L, Vidal J, Bidaud P, Rigaud A. Robot services for elderly with cognitive impairment: Testing usability of graphical user interfaces. Technology and Health Care. 2013;21:217 – 31.
48. Cory-Ann S, Akanksha P, Jenay MB, Mitzner T, Kemp C, Rogers W. Older Adults' Preferences for and Acceptance of Robot Assistance for Everyday Living Tasks. Proceedings of the Human Factors and Ergonomics Society Annual Meeting. 2012;56:153 – 7.
49. Cory-Ann S, Mitzner T, Jenay MB, Akanksha P, Tiffany LC, Kemp C, et al. Domestic Robots for Older Adults: Attitudes, Preferences, and Potential. International Journal of Social Robotics. 2014;6:229–47.
50. Cristina G, Nejat G. The adoption of socially assistive robots for long-term care: During COVID-19 and in a post-pandemic society. Healthcare Management Forum. 2022;35:301 – 9.
51. Cristina G, Nejat G. Investigating Persuasive Socially Assistive Robot Behavior Strategies for Sustained Engagement in Long-Term Care. ArXiv. 2024;abs/2408.14322.
52. Dagioglou M, Andreas L, Franziska K, Doğruz AS, Konstantopoulos S. Interacting with and via mobile devices and mobile robots in an assisted living setting. EAI Endorsed Trans Pervasive Health Technol. 2015;1:e3.
53. Darko EM, Manal K, Gillian L, Tavakoli M. Robotics in Nursing: Protocol for a Scoping Review. JMIR Research Protocols. 2023;12.
54. David P, Alvito P, Eleni C, Samaras G, Dias J. A Study on the Deployment of a Service Robot in an Elderly Care Center. International Journal of Social Robotics. 2018;11:317 – 41.
55. Dekang Z, Qianyi B, Zhongpan Z, Yujie Z, Zhipeng W. Advancing autonomy through lifelong learning: a survey of autonomous intelligent systems. Frontiers in Neurorobotics. 2024;18.
56. Denecke K, Baudoin C. A Review of Artificial Intelligence and Robotics in Transformed Health Ecosystems. Frontiers in Medicine. 2022;9.
57. Dilip G, Ramakrishna G, Sivaram R, Hemalatha S, Pandey RR, Ashim B, et al. Artificial Intelligence-Based Smart Comrade Robot for Elders Healthcare with Strait Rescue System. Journal of Healthcare Engineering. 2022;2022.
58. Dio MJ, Patricia MD, Dion K, Szanton S, Irvin LO. Nursing and human-computer interaction in healthcare robots for older people: An integrative review. International Journal of Nursing Studies Advances. 2022;4.
59. Donghui Z, Xingwang S, Bo S, Zihao Y, Junyou Y, Houdei L, et al. Research status of elderly-care robots and safe human-robot interaction methods. Frontiers in Neuroscience. 2023;17.
60. Edelman L, McConnell E, Kennerly S, Alderden J, Horn S, Yap T. Mitigating the Effects of a Pandemic: Facilitating Improved Nursing Home Care Delivery Through Technology. JMIR Aging. 2020;3.
61. Emel G, Alan H. Perspectives of physicians, nurses, and patients on the use of artificial intelligence and robotic nurses in healthcare. International Nursing Review. 2025;72.
62. Erika Y, Lillian H, Joey W, Wong K, Amanda Y, Jim M, et al. The perceptions of university students on technological and ethical risks of using robots in long-term care homes. Frontiers in Robotics and AI. 2023;10.
63. Esther R, Stephanie L, Sabina M. Service Robots: Emotions of Older Adults in Different Situations. 2020.
64. Fbio C, Ribeiro T, Lopes G, Ribeiro A. Large-Scale Tactile Detection System Based on Supervised Learning for Service Robots Human Interaction. Sensors (Basel, Switzerland). 2023;23.
65. Feng Z, Xin D, Wenli L, Zhihui L, Shih-Chia H. Fidan: a predictive service demand model for assisting nursing home health-care robots. Connection Science. 2023;35.
66. Fiorini L, Raffaele E, Manuele B, Claudio P, Filippo S, Roberta G, et al. Enabling personalised medical support for chronic disease management through a hybrid robot-cloud approach. Autonomous Robots. 2016;41:1263 – 76.

67. Fischinger D, Einramhof P, Papoutsakis K, Wohlkinger W, Mayer P, Panek P, et al. Hobbit, a care robot supporting independent living at home: First prototype and lessons learned. Robotics Auton Syst. 2016;75:60–78.
68. Friedrich O, Selin G, Seifert J, Sebastian S. Value change through information exchange in human-machine interaction. Prometheus. 2022.
69. Gabriele V, Anne-Sophie LK. Robotic devices and ICT in long-term care in Japan: Their potential and limitations from a workplace perspective. Contemporary Japan. 2021;35:270 – 90.
70. Gabriella C, Riccardo De B, Francesca F, Andrea O, Umbrico A, Cesta A. AI and robotics to help older adults: Revisiting projects in search of lessons learned. Paladyn, Journal of Behavioral Robotics. 2021;12:356 – 78.
71. Gang F, Falin W, Wei L, Libin X, Wenxiang Z, Man T, et al. Artificial Intelligence in Chronic Disease Management for Aging Populations: A Systematic Review of Machine Learning and NLP Applications. International Journal of General Medicine. 2025;18:3105 – 15.
72. Gasteiger N, Kate L, Mikaela L, Broadbent E. Friends from the Future: A Scoping Review of Research into Robots and Computer Agents to Combat Loneliness in Older People. Clinical Interventions in Aging. 2021;16:941 – 71.
73. Gianluca B, Antonini A, Motta E. Robots for Elderly Care in the Home: A Landscape Analysis and Co-Design Toolkit. International Journal of Social Robotics. 2021;14:657 – 81.
74. Gliesche P, Tobias K, Pfingsthorn M, Drolshagen S, Kowalski C, Hein A. Kinesthetic Device vs. Keyboard/Mouse: A Comparison in Home Care Telemanipulation. Frontiers in Robotics and AI. 2020;7.
75. Gonzlez-Gonzlez C, Violant-Holz V, Rosa Mara G-I. Social Robots in Hospitals: A Systematic Review. Applied Sciences. 2021.
76. Grace TB, Jenna-Marie G, Joseph T, Stephanie Tulk J. A systematic review of collaborative robots for nurses: where are we now, and where is the evidence? Frontiers in Robotics and AI. 2024;11.
77. Graf P, Christian Snderskov Z-F, Lakshadeep N, Kevin L, Emanuela M, Eva H, et al. Distributed agency in HRI-an exploratory study of a narrative robot design. Frontiers in Robotics and AI. 2024;11.
78. Guan-Yu L, Ji-Huan R, Yi-Shun W. Factors Affecting Family Caregivers' Behavioral Intention to Use Socially Assistive AI Robots for Elderly Care Within Their Own Home Environment. International Journal of Human–Computer Interaction. 2023;40:7286 – 96.
79. Haubold A, Lisa O, Franziska B. Introducing service robotics in inpatient geriatric care—a qualitative systematic review from a human resources perspective. Gruppe Interaktion Organisation Zeitschrift f \ u r Angewandte Organisationspsychologie (GIO). 2020;51:259 – 71.
80. Hebah E, Ghada Al A, Rawan A, Reema A, Samar A. Telepresence Robot System for People with Speech or Mobility Disabilities. Sensors (Basel, Switzerland). 2022;22.
81. Hebesberger D, Krtner T, Gisinger C, Pripfl J. A Long-Term Autonomous Robot at a Care Hospital: A Mixed Methods Study on Social Acceptance and Experiences of Staff and Older Adults. International Journal of Social Robotics. 2017;9:417 – 29.
82. Heesun S, Jeon C. The Robotic Multi-Care Network: A Field Study of a "Robot Grandchild" in South Korea. East Asian Science, Technology and Society: An International Journal. 2024;18:177 – 95.
83. Hocheol L, Min Ah C, Kim H, Nam E. The Effect of Cognitive Function Health Care Using Artificial Intelligence Robots for Older Adults: Systematic Review and Meta-analysis. JMIR Aging. 2022;5.
84. Hojjat A, Mahoor M, Rohola Z, Jarid S, Qualls S. Artificial Emotional Intelligence in Socially Assistive Robots for Older Adults: A Pilot Study. IEEE Transactions on Affective Computing. 2022;14:2020–32.
85. Honghao L, Geng Y, Huiying Z, Xiaoyan H, Huayong Y, Zhibo P. Teleoperation of Collaborative Robot for Remote Dementia Care in Home Environments. IEEE Journal of Translational Engineering in Health and Medicine. 2020;8.
86. Hoppe J, Tuisku O, Rose-Marie J-P, Satu P, Hennala L, Gustafsson C, et al. When do individuals choose care robots over a human caregiver? Insights from a laboratory experiment on choices under uncertainty. Computers in Human Behavior Reports. 2022.
87. Hudson J, Marta O, Huňady J. People's Attitudes to Robots in Caring for the Elderly. International Journal of Social Robotics. 2017;9:199–210.
88. Hung L, Wong J, Ren H, Yong Z, Fu J, Jim M, et al. The Impact of Telepresence Robots on Family Caregivers and Residents in Long-Term Care. International Journal of Environmental Research and Public Health. 2025;22.
89. Ida F, Hariyati RT, Etty R. Pemanfaatan Teknologi Robot dalam Peningkatan Kualitas Tidur dan Pencegahan Jatuh pada Lansia. Journal of Telenursing (JOTING). 2023.

90. Imojean N, Sanetta HJdT, Meryl L. The use of technology to promote meaningful engagement for adults with dementia in residential aged care: a scoping review. International Psychogeriatrics. 2019;32:913 – 35.
91. In-Jin Y, do-Hyung P, Othelia EunKyung L, Albert P. Investigating Older Adults' Use of a Socially Assistive Robot via Time Series Clustering and User Profiling: Descriptive Analysis Study. JMIR Formative Research. 2024;8.
92. Janika L, Kadi L, Kristel M, Katrin K. Evaluating Social Assistive Robots in Clinical Nursing Care: Mixed Method Pilot Study on Health Care Workers' Perceptions and Adoption. JMIR Nursing. 2024;8.
93. Jari P, Melkas H, Arto L, Satu P. Could robots strengthen the sense of autonomy of older people residing in assisted living facilities?—A future-oriented study. Ethics and Information Technology. 2019;22:151–62.
94. Jeongeun K, Sukwha K, Kim S, Euehun L, Heo Y, Cheol-Yong H, et al. Companion robots for older adults: Rodgers' evolutionary concept analysis approach. Intelligent Service Robotics. 2021;14:729 – 39.
95. Jiajun S, Bin X, Mingtao P, Yunde J. A low-cost tele-presence wheelchair system. 2016 IEEE/RSJ International Conference on Intelligent Robots and Systems (IROS). 2016:2452–7.
96. Jiaying W. The Development of Elderly Care Robot and Current Challenges for Functional Design. IOP Conference Series: Materials Science and Engineering. 2020;926.
97. Jing F, Mion L, Linda B, Ullal A, Newhouse P, Sarkar N. SAR-Connect: A Socially Assistive Robotic System to Support Activity and Social Engagement of Older Adults. IEEE Transactions on Robotics. 2022;38:1250–69.
98. Jing L, Xingang W, Jiaqi Z. Investigating Elderly Individuals' Acceptance of Artificial Intelligence (AI)-Powered Companion Robots: The Influence of Individual Characteristics. Behavioral Sciences. 2025;15.
99. Joannes Paulus Tolentino H. Compassionate Care with Autonomous AI Humanoid Robots in Future Healthcare Delivery: A Multisensory Simulation of Next-Generation Models. Biomimetics. 2024;9.
100. Johnson M, Sobrepera MJ, Kina E, Rochelle JM. Design of an Affordable Socially Assistive Robot for Remote Health and Function Monitoring and Prognostication. International Journal of Prognostics and Health Management. 2023.
101. Jong-Wook K, Young-Lim C, Sang-Hyun J, Jeong-Hui H. A Care Robot with Ethical Sensing System for Older Adults at Home. Sensors (Basel, Switzerland). 2022;22.
102. Jordan A, Al-Hindawi A, Tiffany N, Vizcaychipi M. Scoping review on the use of socially assistive robot technology in elderly care. BMJ Open. 2018;8.
103. Joschka H, Johanna H, Vollmann J. Aging between Participation and Simulation. 2020.
104. Juan F, Matari M. Socially Assistive Robot Exercise Coach: Motivating Older Adults to Engage in Physical Exercise. 2012.
105. Julia H, Selcan O, Artur L, Ayşegl D. A Systematic Literature Review on the Use of Social Robots in Elderly Care. Proceedings of the 35th Australian Computer-Human Interaction Conference. 2023.
106. Kang H, In Soon K, Makimoto K, Yamakawa M. Nurses' perception towards care robots and their work experience with socially assistive technology during COVID-19: A qualitative study. Geriatric Nursing (New York, Ny). 2023;50:234 – 9.
107. Katie T, Elliott H, Bo X. Robots in Senior-Living Facilities: A Systematic Literature Review. Innovation in Aging. 2022;6:70 –
108. Katie T, Liu J, Elliott H, Bo X. Nursing Staff's Attitudes, Needs, and Preferences for Care Robots in Assisted Living Facilities: A Systematic Literature Review. Companion of the 2024 ACM/IEEE International Conference on Human-Robot Interaction. 2024.
109. Katie T, Liu J, Elliott H, Bo X. Nursing Staff's Perspectives of Care Robots for Assisted Living Facilities: Systematic Literature Review. JMIR Aging. 2024;7.
110. Katie T, Rachel T, Bo X, Elliott H. Robots in Assisted Living Facilities: Scoping Review. JMIR Aging. 2022;6.
111. Kbra Beliz B, Golnaz LA, Viktoria H, Franziska Laporte U, Meiland F, Teupen S, et al. Can technology impact loneliness in dementia? A scoping review on the role of assistive technologies in delivering psychosocial interventions in long-term care. Disability and Rehabilitation: Assistive Technology. 2021;18:1107 – 19.
112. Ke C, Lou V, Tan K, Man-Yi W, Lai-Lok C. Changes in technology acceptance among older people with dementia: the role of social robot engagement. International journal of medical informatics. 2020;141:104241.
113. Kenji K, Tatsuya Y, Aimoto K, Kenji S, Itoh N, Izumi K. Reduction of multiple-caregiver assistance through the long-term use of a transfer support robot in a nursing facility. Assistive Technology. 2022;35:271 – 8.
114. Kenji K, Tatsuya Y, Aimoto K, Kenji S, Itoh N, Izumi K. A rise-assisting robot extends life space and improves facial expressions of nursing home residents. BMC Health Services Research. 2022;22.

115. Kenji K, Yasunari H, Aimoto K, Masaki K, Koki K, Tatsuya Y, et al. Electrocardiogram and respiration recordings show a reduction in the physical burden on professional caregivers when performing care tasks with a transfer support robot. Assistive Technology. 2024;37:185 – 93.
116. Kentaro W, Miwa H, Tomoko W, Kajitani I. Adopting the service system view toward successful implementation of assistive technologies. Geriatrics \& Gerontology International. 2024;24:74 – 80.
117. Khaksar W, Diana S, Bygrave L, Trresen J. Robotics in Elderly Healthcare: A Qualitative Analysis of 20 Recent European Research Projects. ACM Transactions on Human-Robot Interaction. 2023;14:1 – 38.
118. Khan Z, Afifa S, Chang Won L. Robotics Utilization for Healthcare Digitization in Global COVID-19 Management. International Journal of Environmental Research and Public Health. 2020;17.
119. Kipnis E, McLeay F, Grimes A, Stevienna de S, Stephen P. Service Robots in Long-Term Care: A Consumer-Centric View. Journal of Service Research. 2022;25:667 – 85.
120. Ko J, Kong Y, Kyeong‐Hee C, Lee C, Hyunji K, Hong J, et al. Comparison of the Physical Care Burden on Formal Caregivers between Manual Human Care Using a Paper Diaper and Robot-Aided Care in Excretion Care. International Journal of Environmental Research and Public Health. 2023;20.
121. Koceska N, Koceski S, Zobel PB, Trajkovik V, Garcia N. A Telemedicine Robot System for Assisted and Independent Living. Sensors (Basel, Switzerland). 2019;19.
122. Koen S, Matthijs HJS, Sil B, Jurgen B, Guido E, Lars P. Hello, is Someone There? A Case Study for Using a Social Robot in Dementia Care. 2021.
123. Krtner T, Alexandra S, Daliah B-K, Gisinger C, Andreas H, Lammer L, et al. How Social Robots Make Older Users Really Feel Well - A Method to Assess Users' Concepts of a Social Robotic Assistant. 2012.
124. Lambr MMR, Est R. A Literature Review on New Robotics: Automation from Love to War. International Journal of Social Robotics. 2015;7:549 – 70.
125. Laura P, Jun J, Masood D, Martin J. A Quantitative Analysis of Activities of Daily Living: Insights into Improving Functional Independence with Assistive Robotics. 2022 International Conference on Robotics and Automation (ICRA). 2021:6999–7006.
126. Laura S, Bilge M. Designing for Caregiving: Integrating Robotic Assistance in Senior Living Communities. Proceedings of the 2022 ACM Designing Interactive Systems Conference. 2022.
127. Laura S, David JP, Mark R, Laura MH. Considerations for End-User Development in the Caregiving Domain. ArXiv. 2024;abs/2402.17836.
128. Laura S, Emmanuel S, Bilge M. Factors that Affect Personalization of Robots for Older Adults. ArXiv. 2024;abs/2402.17769.
129. Leonardsen A, Camilla H, Helgesen A, Bth C, Lilliana Andrea Del B, Grndahl VA. The Use of Robotic Technology in the Healthcare of People above the Age of 65—A Systematic Review. Healthcare. 2023;11.
130. Leung A, Zhao I, Shuanglan L, Terence KL. Exploring the Presence of Humanoid Social Robots at Home and Capturing Human-Robot Interactions with Older Adults: Experiences from Four Case Studies. Healthcare. 2022;11.
131. Li-Chin L, Shao-Huan L, Yen-Ping H, Long‐Yau L, Lan S, Jong-Chen C. Effectiveness of Companion Robot Care for Dementia: A Systematic Review and Meta-Analysis. Innovation in Aging. 2021;5.
132. Lihui P, Moyle W, Cindy J, Todorovic M. The Effectiveness of Social Robots for Older Adults: A Systematic Review and Meta-Analysis of Randomized Controlled Studies. The Gerontologist. 2018;59:e37–e51.
133. Lili L, Eleni S, Nikolaidis I, Antonio Miguel C, Rincn A. Smart homes and home health monitoring technologies for older adults: A systematic review. International journal of medical informatics. 2016;91:44–59.
134. Lillian H, Abdul-Fatawu A, Albin S, Wong K, Ren L, Rachel X, et al. Students perspectives on the development and deployment of an AI-enabled service robot in long-term care. Journal of Rehabilitation and Assistive Technologies Engineering. 2025;12.
135. Lillian H, Hiro I, Joey W. Lovot Robot as Companions for Older Adults in Long-Term Care. Innovation in Aging. 2023;7:1077 –
136. Lillian H, Irene C, Joey W, Yong Z, Wong K, Sadaf M-K, et al. Facilitators and barriers to codesigning social robots with older adults living with dementia: A scoping review. Digital Health. 2025;11.
137. Lillian H, Jim M, Jennifer P, Annette B, Joey W. Technological risks and ethical implications of using robots in long-term care. Journal of Rehabilitation and Assistive Technologies Engineering. 2022;9.
138. Lillian H, Ren H, Wong K, Katharine D, Arisa K. Feasibility and Acceptability of Implementing an Ai-Enabled Service Robot in Long-Term Care. Innovation in Aging. 2024;8:38 –

139. Lillian H, Wong K, Joey W, Juyoung P, Hadil A, Yong Z, et al. Healthcare Workers' Perspectives on Ai-Enabled Robots Use in Long-Term Care: A Scoping Review. Innovation in Aging. 2023;7:1053 –
140. Lillian H, Wong K, Joey W, Juyoung P, Hossein M, Hui Z. Facilitators and barriers to using AI-enabled robots with older adults in long-term care from staff perspective: a scoping review protocol. BMJ Open. 2023;13.
141. Lin W, Jia C, Da-Young J. Factors Contributing to Korean Older Adults' Acceptance of Assistive Social Robots. Electronics. 2021.
142. Linda S, Dag Tomas Sagen J, Melkas H, Johnsen HM. User Acceptance of a Home Robotic Assistant for Individuals With Physical Disabilities: Explorative Qualitative Study. JMIR Rehabilitation and Assistive Technologies. 2024;12.
143. Linner T, Pan W, Georgoulas C, Georgescu B, Gttler J, Bock T. Co-adaptation of Robot Systems, Processes and In-house Environments for Professional Care Assistance in an Ageing Society. Procedia Engineering. 2014;85:328–38.
144. Lisa O, Franziska B, Rdiger von der W, Michael D. Service Robots in Nursing Homes (SeRoNu): a holistic model of influencing factors. Gruppe Interaktion Organisation Zeitschrift f \ u r Angewandte Organisationspsychologie (GIO). 2022;53:285 – 93.
145. Lisa T, Khosla R. Using Social Robots in Health Settings: Implications of Personalization on Human-Machine Communication. Communicatio. 2016;5:1–17.
146. Louie W, Jacob L, Vaquero T, Nejat G. A focus group study on the design considerations and impressions of a socially assistive robot for long-term care. The 23rd IEEE International Symposium on Robot and Human Interactive Communication. 2014:237–42.
147. Lu V, Jochen W, Kunz W, Stefanie P, Thorsten G, Antje M, et al. Service robots, customers and service employees: what can we learn from the academic literature and where are the gaps? Journal of Service Theory and Practice. 2020.
148. Luis Cobo H, Pablo V, Zalama E, Gmez-Garca-Bermejo J, Jos Mara D, Beatriz Vielba G. Development and Usability Validation of a Social Robot Platform for Physical and Cognitive Stimulation in Elder Care Facilities. Healthcare. 2021;9.
149. Lukkien D, Nap H, Buimer H, Peine A, Boon W, Ket J, et al. Toward Responsible Artificial Intelligence in Long-Term Care: A Scoping Review on Practical Approaches. The Gerontologist. 2021;63:155 – 68.
150. Malin A, Linda N, Eide H, Fagerstrm L. Humanoid robots in the care of older persons: A scoping review. Assistive Technology. 2021;34:518 – 26.
151. Malin A, Linda N, Eide H, Rauhala A, Fagerstrm L. Attitudes toward the use of humanoid robots in healthcare—a cross-sectional study. Ai \& Society. 2021;37:1739–48.
152. Marcel H, Krse B, Evers V, Wielinga B. Assessing Acceptance of Assistive Social Agent Technology by Older Adults: the Almere Model. International Journal of Social Robotics. 2010;2:361–75.
153. Marcus P, David R, Iversen C. Caregivers' use of robots and their effect on work environment – a scoping review. Journal of Technology in Human Services. 2021;40:251 – 77.
154. Margo AMvK, Hoorn J, Konijn E. Healthcare Students' Ethical Considerations of Care Robots in The Netherlands. Applied Sciences. 2018.
155. Maria K, Fotios L, Akilesh R, Christos S, Harish Ram N, Chaitanya K, et al. A Survey of Robots in Healthcare. Technologies. 2021.
156. Marian RB, Willoughby L, Banks W. Animal-assisted therapy and loneliness in nursing homes: use of robotic versus living dogs. Journal of the American Medical Directors Association. 2008;9 3:173–7.
157. Maribel P, Boulay M, Jouen F, Rigaud A. "Are we ready for robots that care for us?" Attitudes and opinions of older adults toward socially assistive robots. Frontiers in Aging Neuroscience. 2015;7.
158. Marina M, Sander LH, Eva C, Hall J, Miranda L, Atzema C, et al. A qualitative study on the perspectives of patients and providers on the integration of geriatric personal support workers for improving quality of care in the emergency department. Canadian Journal of Emergency Medicine. 2025;27:830 – 9.
159. Martin C, Staffa M. Special Issue on Social Robots for Human Well-Being. Robotics. 2025;14:37.
160. Martina a, Mahr D, Gaby O-S. Value of social robots in services: social cognition perspective. Journal of Services Marketing. 2019.
161. Masala G, Ioanna G. Artificial Intelligence and Assistive Robotics in Healthcare Services: Applications in Silver Care. International Journal of Environmental Research and Public Health. 2025;22.

162. Melkas H, Hennala L, Satu P, Ville K. Impacts of robot implementation on care personnel and clients in elderly-care institutions. International journal of medical informatics. 2019;134:104041.
163. Mikaela L, Craig JS, Ahn H, MacDonald B, Kathy P, Johanson D, et al. Developing assistive robots for people with mild cognitive impairment and mild dementia: a qualitative study with older adults and experts in aged care. BMJ Open. 2019;9.
164. Mio N, Nihei M, Kato N, Takenobu I. Impact of the introduction of a verbal socially assistive robot on the relationship between older people and their caregivers in a nursing home. SN Applied Sciences. 2020;2.
165. Miura C, Sinan C, Saiki S, Masahide N, Yasuda K. Assisting Personalized Healthcare of Elderly People: Developing a Rule-Based Virtual Caregiver System Using Mobile Chatbot. Sensors (Basel, Switzerland). 2022;22.
166. Miyoung C, Dohyung K, Minsu J, Jaehong K, Woo-han Y, Youngwoo Y, et al. Evaluating Human-Care Robot Services for the Elderly: An Experimental Study. International Journal of Social Robotics. 2024;16:1561 – 87.
167. Mois G, Jenay MB. The Role of Healthcare Robotics in Providing Support to Older Adults: a Socio-ecological Perspective. Current Geriatrics Reports. 2020;9:82 – 9.
168. Mousumi K, Sume A, Iffath M, Fakhrul I, Samsul C, Nayla Mehereen A, et al. The Potential of Artificial Intelligence in Unveiling Healthcare's Future. Cureus. 2024;16.
169. Nada F, Sherratt R, Elhajj I. Directing and Orienting ICT Healthcare Solutions to Address the Needs of the Aging Population. Healthcare. 2021;9.
170. Nejat G, Amos JZ, Cristina G, Hila S-K. Adoption of assistive technologies in long-term care homes: What the pandemic has taught us. Healthcare Management Forum. 2024;37:418 – 22.
171. Neziha A, Annica K, Loutfi A. The Influence of Feedback Type in Robot-Assisted Training. Multimodal Technol Interact. 2019;3:67.
172. Nghia Chi N, Masami S. Issues in applications of nursing care robots, and in the training of care workers in their use in Japan. Frontiers in Medicine. 2025;12.
173. Nina J, Pedersen I, Rokstad A, Ihlebk C. Effects on Symptoms of Agitation and Depression in Persons With Dementia Participating in Robot-Assisted Activity: A Cluster-Randomized Controlled Trial. Journal of the American Medical Directors Association. 2015;16 10:867–73.
174. Niyati D, Ashish PA. Artificial Intelligence With Robotics in Healthcare: A Narrative Review of Its Viability in India. Cureus. 2023;15.
175. Noel M, Abbas S, Elhajj I, Daniel CA. Robotics in Nursing: A Scoping Review. Journal of Nursing Scholarship. 2018;50:590–600.
176. Norma C, Yeong C, Patrick P. Reducing the Burdens of Paid Caregivers of Older Adults by Using Assistive Technology: A Scoping Review. Western Journal of Nursing Research. 2024;46:315 – 26.
177. Nourhan A, Yue H. Human Factors Considerations for Quantifiable Human States in Physical Human–Robot Interaction: A Literature Review. Sensors (Basel, Switzerland). 2023;23.
178. Nria V-P, Oriol B-A, Miquel D. Robots in Healthcare? What Patients Say. International Journal of Environmental Research and Public Health. 2021;18.
179. Okamoto S, Komamura K. Towards universal health coverage in the context of population ageing: a narrative review on the implications from the long-term care system in Japan. Archives of Public Health. 2022;80.
180. Olatunji SA, James SS, Adam S, Yao-Lin T, April EP, Harshal PM, et al. Robotic support for older adults with cognitive and mobility impairments. Frontiers in Robotics and AI. 2025;12.
181. Papadopoulos I, Koulouglioti C, Lazzarino R, Sheila A. Enablers and barriers to the implementation of socially assistive humanoid robots in health and social care: a systematic review. BMJ Open. 2020;10.
182. Papadopoulos I, Koulouglioti C, Sheila A. Views of nurses and other health and social care workers on the use of assistive humanoid and animal-like robots in health and social care: a scoping review. Contemporary Nurse. 2018;54:425 – 42.
183. Paul Notger L, Camille G, Daniel F, Corinna D, Felix B, Thorsten S. Health Care Social Robots in the Age of Generative AI: Protocol for a Scoping Review. JMIR Research Protocols. 2024;14.
184. Plohl N, Flis V, Bergauer A, Kobilica N, Tadej K, Samo H, et al. A protocol on the effects of interactive digital assistance on engagement and perceived quality of care of surgery patients and self-efficacy and workload of staff. Frontiers in Medicine. 2022;9.
185. Pouyan A, Panchea A, François F. A Review on the Use of Mobile Service Robots in Elderly Care. Robotics. 2022;11:127.

186. Priyesh T. Some Non-technology Implications for Wider Application of Robots to Assist Older People. 2009.
187. Rachel ES, Rogers W. Older Adults' Perceptions of Supporting Factors of Trust in a Robot Care Provider. J Robotics. 2018;2018:6519713 1– 11.
188. Ralf V, Anna D, Frijns H, Laura V, Katharina B, Christopher F. From care practices to speculative vignettes–design considerations for robots in good care. Frontiers in Robotics and AI. 2024;11.
189. Rana I, Arshia K. Perceptions of Humanoid Robots in Caregiving: A Study of Skilled Nursing Home and Long Term Care Administrators. ArXiv. 2024;abs/2401.02105.
190. Rebecca QS, MacDonald B, Jayawardena C, Wegner D, Broadbent E. Does the Robot Have a Mind? Mind Perception and Attitudes Towards Robots Predict Use of an Eldercare Robot. International Journal of Social Robotics. 2014;6:17–32.
191. Rebecca R, Bri D, Brian S. A Grounded Observer Framework for Establishing Guardrails for Foundation Models in Socially Sensitive Domains. ArXiv. 2024;abs/2412.18639.
192. Reddy S. Use of Artificial Intelligence in Healthcare Delivery. eHealth - Making Health Care Smarter. 2018.
193. Ren H, Wong K, Albin S, Kayoung L, Shambhavi A, Julia B, et al. Feasibility and Acceptability of Deploying a Collaborative Service Robot in Long-Term Care: Staff Experiences. Electronics. 2025.
194. Ren L, Wong K, Joey W, Sarah K, Annette B, Jim M, et al. Working with a robot in hospital and long-term care homes: staff experience. BMC Nursing. 2024;23.
195. Reza K, Sedighadeli S, Khosla R, Mei-Tai C. Socially Assistive Robots in Elderly Care: A Mixed-Method Systematic Literature Review. International Journal of Human-Computer Interaction. 2014;30:369 – 93.
196. Rhian CP, Madison RS, Carolyn MA, Naomi TF. How can robots facilitate physical, cognitive, and social engagement in skilled nursing facilities? Frontiers in Aging. 2024;5.
197. Ribeiro T, Fernando G, Ines G, Lopes G, Ribeiro A. CHARMIE: A Collaborative Healthcare and Home Service and Assistant Robot for Elderly Care. Applied Sciences. 2021.
198. Riek L. Healthcare robotics. Communications of the ACM. 2017;60:68 – 78.
199. Rigaud A, Dacunha S, Harzo C, Lenoir H, Imad S, Piccoli M, et al. Implementation of socially assistive robots in geriatric care institutions: Healthcare professionals' perspectives and identification of facilitating factors and barriers. Journal of Rehabilitation and Assistive Technologies Engineering. 2024;11.
200. Roberto Pinillos H, Samuel M-P, Gmez-Garca-Bermejo J, Eduardo Zalama C. Towards the acceptance of care robots by senior users. Eighth International Conference on Technological Ecosystems for Enhancing Multiculturality. 2020.
201. Robinson H, MacDonald B, Broadbent E. The Role of Healthcare Robots for Older People at Home: A Review. International Journal of Social Robotics. 2014;6:575–91.
202. Roger B, Gelderblom G, Jonker P, Witte LDd. Socially assistive robots in elderly care: a systematic review into effects and effectiveness. Journal of the American Medical Directors Association. 2012;13 2:114–20.e1.
203. Rogers W, Bayles M. Design and Use of Robots to Assist Older Adults with Healthcare Tasks. Innovation in Aging. 2019;3:S32 – S.
204. Rogers W, Kadylak T, Bayles M. Maximizing the Benefits of Participatory Design for Human–Robot Interaction Research With Older Adults. Human Factors: The Journal of Human Factors and Ergonomics Society. 2021;64:441 – 50.
205. Rose-Marie J-P, Thommes K, Hoppe J, Tuisku O, Hennala L, Satu P, et al. Care Robot Orientation: What, Who and How? Potential Users' Perceptions. International Journal of Social Robotics. 2020;12:1103 – 17.
206. Ruan Y, Che R, Kodate N, Donnelly S, Yiwen S, Xiaoting L, et al. A Scoping Review on Long‐Term Care Workers' Perceptions of Robot‐Assisted Care for Older Adults in Long‐Term Care Facilities. Journal of Advanced Nursing. 2025;81:8409‐31.
207. Sabelli A, Kanda T, Hagita N. A conversational robot in an elderly care center: An ethnographic study. 2011 6th ACM/IEEE International Conference on Human-Robot Interaction (HRI). 2011:37–44.
208. Samuel AO, Husna H, Kenneth B, Uppalapati N, Girish K, Wendy AR. Investigating Older Adults' Perspectives on Telehealth Robotics. Innovation in Aging. 2022;6:841 –
209. Samuel AO, Vy N, Maya C, Edsinger A, Charles K, Rogers W, et al. Immersive participatory design of assistive robots to support older adults. Ergonomics. 2024;67:717 – 31.
210. Sapci A, Sapci H. Innovative Assisted Living Tools, Remote Monitoring Technologies, Artificial Intelligence-Driven Solutions, and Robotic Systems for Aging Societies: Systematic Review. JMIR Aging. 2019;2.

211. Sara S, Benjamin KPW. Use of technology and social media in dementia care: Current and future directions. World Journal of Psychiatry. 2021;11:109 – 23.
212. Satu K-U, Vaismoradi M, Katajisto J, Kangasniemi M, Riitta T. Effect of robot for medication management on home care professionals' use of working time in older people's home care: a non-randomized controlled clinical trial. BMC Health Services Research. 2023;23.
213. Sebastian S, Christian T, Kok C, Hofstede B, Askari SI, Hoesel TV, et al. Huggable integrated socially assistive robots: exploring the potential and challenges for sustainable use in long-term care contexts. Frontiers in Robotics and AI. 2025;12.
214. Shuai Y, Simon C, Reeva ML, Jenny W. Ethical Design of Social Robots in Aged Care: A Literature Review Using an Ethics of Care Perspective. International Journal of Social Robotics. 2023;15:1637–54.
215. Silvana C. Technologies of Care: Robot Caregivers in Science and Fiction. Humanities. 2023.
216. Silvera-Tawil D. Robotics in Healthcare: A Survey. SN Computer Science. 2024;5:1–19.
217. Smola P, Iwona M, Wojcieszko M, Zwierczyk U, Mateusz K, Elzbieta R, et al. Attitudes toward artificial intelligence and robots in healthcare in the general population: a qualitative study. Frontiers in Digital Health. 2025;7.
218. Souren P, Nejat G. The Future of Intelligent Healthcare: A Systematic Analysis and Discussion on the Integration and Impact of Robots Using Large Language Models for Healthcare. ArXiv. 2024;abs/2411.03287.
219. Srikanta P, Mohapatra A, Ramasamy S, Sanjana A. Artificial Intelligence (AI) and Robotics in Elderly Healthcare: Enabling Independence and Quality of Life. Cureus. 2023;15.
220. Stra H, Fosch-Villaronga E. Healthcare Digitalisation and the Changing Nature of Work and Society. Healthcare. 2021;9.
221. Su-Jung N, Eun-Young P. Effectiveness of Robot Care Intervention and Maintenance for People with Dementia: A Systematic Review and Meta-Analysis. Innovation in Aging. 2024;9.
222. Suwa S, Tsujimura M, Hiroo I, Kodate N, Mina I, Atsuko S, et al. Home-care Professionals' Ethical Perceptions of the Development and Use of Home-care Robots for Older Adults in Japan. International Journal of Human–Computer Interaction. 2020;36:1295 – 303.
223. Svetlana Gennadevna K, Grigoriev AV, Kiseleva E, Polyakova A, Barinov S. Artificial intelligence in the service of man: medical, social and economic aspects. Laplage Em Revista. 2021.
224. Szczepura A, Nomura T, Wild D. The Future of Robots in a Super-Aged Society. 2020.
225. Takayanagi K, Takahiro K, Shibata T. Comparison of Verbal and Emotional Responses of Elderly People with Mild/Moderate Dementia and Those with Severe Dementia in Responses to Seal Robot, PARO. Frontiers in Aging Neuroscience. 2014;6.
226. Takeshi H, Qinghua S, Takuya F, Takawaki R, Eriko A, Kenji N, et al. Emotional Speech Synthesis for Companion Robot to Imitate Professional Caregiver Speech. ArXiv. 2021;abs/2109.12787.
227. Tan S, Araz T, Abhas T. Tensions and antagonistic interactions of risks and ethics of using robotics and autonomous systems in long-term care. Technological Forecasting and Social Change. 2021;167:120686.
228. Tanioka T, Feni B, Tomoya Y, Osaka K, Locsin R, King B, et al. The experience of older persons with mental health conditions who interact with healthcare robots and nurse intermediaries: The qualitative case studies. Belitung Nursing Journal. 2021;7:346 – 53.
229. Tapus A, Cristian T, Matari M. The use of socially assistive robots in the design of intelligent cognitive therapies for people with dementia. 2009 IEEE International Conference on Rehabilitation Robotics. 2009:924–9.
230. Taslim SM, Uddin R. Enhancing Human-Robot Interaction in Healthcare: A Study on Nonverbal Communication Cues and Trust Dynamics with NAO Robot Caregivers. ArXiv. 2025;abs/2503.16469.
231. Tatsuya Y, Kenji K, Aimoto K, Izumi K. Robotic Care Equipment Improves Communication between Care Recipient and Caregiver in a Nursing Home as Revealed by Gaze Analysis: A Case Study. International Journal of Environmental Research and Public Health. 2024;21.
232. Tatsuya Y, Kenji K, Shohei T, Nobuaki M, Aimoto K, Itoh N, et al. Investigating proficiency using a lift-type transfer support device for effective care: comparison of skilled and unskilled nursing homes. Disability and Rehabilitation: Assistive Technology. 2022;19:841 – 50.
233. Tianyang H, Haitao L. Acceptability of Robots to Assist the Elderly by Future Designers: A Case of Guangdong Ocean University Industrial Design Students. Sustainability. 2019.
234. Tijs V, Bernadette Dierckx de C, Gastmans C. How do older adults experience and perceive socially assistive robots in aged care: a systematic review of qualitative evidence. Aging \& Mental Health. 2018;22:149 – 67.

235. Tijs V, Kevin D, Gastmans C. Older adults' experiences with and perceptions of the use of socially assistive robots in aged care: A systematic review of quantitative evidence. Archives of gerontology and geriatrics. 2021;95:104399.
236. Toshiaki T, Shiho H, Tsuneki N, Sachiyo F, Naoki S, Tetsuhiko H, et al. The Long-Term Use of Communication Robots by Users of Visiting Healthcare Services: Development of an Integrated Vital Signs Measurement System. Cureus. 2025;17.
237. Toshiharu I, Iijima K, Kunio N, Yu C. Detailed Analysis of Responses from Older Adults through Natural Speech: Comparison of Questions by AI Agents and Humans. International Journal of Environmental Research and Public Health. 2024;21.
238. Tuisku O, Rose-Marie J-P, Hoppe J, Satu P, Hennala L, Thommes K, et al. Assistant nurses and orientation to care robot use in three European countries. Behaviour \& Information Technology. 2022;42:758 – 74.
239. Tuisku O, Satu P, Hennala L, Melkas H. Robots do not replace a nurse with a beating heart. Inf Technol People. 2019;32:47–67.
240. Tuuli T. Uncertainties about accepting care robots. Frontiers in Digital Health. 2023;5.
241. Tuuli T, Lina Van A, Srkikoski T, Atte O. Finnish healthcare professionals' attitudes towards robots: Reflections on a population sample. Nursing Open. 2018;5:300 – 9.
242. Valeria M, Evonne M, Lee M, Glenda C. Human–robot interactions and experiences of staff and service robots in aged care. Scientific Reports. 2025;15.
243. Ware C, Rigaud AS, Lauriane B, Souad D, Dacunha S, Lenoir H, et al. Development of an ethical framework for the use of social robots in the care of individuals with major neurocognitive disorders: a qualitative study. BMC Geriatrics. 2025;25.
244. Weng W, Han-Pang H, Yu-Lin Z, Chun-Yeon L. Development of a Visual Perception System on a Dual-Arm Mobile Robot for Human-Robot Interaction. Sensors (Basel, Switzerland). 2022;22.
245. Wenting J, Ruzhen L, Yumei S, Maiping Y, Yueheng L, Hua C, et al. A Networked Intelligent Elderly Care Model Based on Nursing Robots to Achieve Healthy Aging. Research. 2025;8.
246. Wong K, Lillian H, Joey W, Juyoung P, Hadil A, Yong Z, et al. Adoption of Artificial Intelligence–Enabled Robots in Long-Term Care Homes by Health Care Providers: Scoping Review. JMIR Aging. 2023;7.
247. Xiuli C, Chen Y, Xiaomiao G, Guochen Z, Guanbin R. Enhancing Value Co-Creation in Wenzhou's Elderly Care Networks:Mathematical Fusion Algorithms for Multi-Sensor Integration in Social Robotics. Proceedings of the 2023 9th International Conference on Robotics and Artificial Intelligence. 2023.
248. Xucong H, Song T. Effects of Robot Animacy and Emotional Expressions on Perspective-Taking Abilities: A Comparative Study across Age Groups. Behavioral Sciences. 2023;13.
249. Yanling D, Xiaolan Z. Advancements in AI-driven multilingual comprehension for social robot interactions: An extensive review. Electronic Research Archive. 2023.
250. Yilin W, Hongxiu C, Hong C, Jing Q, Rui H, Chaobin Z, et al. The attitudes and acceptance of functional assistive robots among older adults with disabilities: a mixed-methods study. Innovation in Aging. 2025;9.
251. Yingwei G, Yingjian Y, Yang L, Qiang L, Fengqiu C, Mengting F, et al. Development Status and Multilevel Classification Strategy of Medical Robots. Electronics. 2021;10:1278.
252. Yizhu L, Nan L, Effati M, Nejat G. Dances with Social Robots: A Pilot Study at Long-Term Care. Robotics. 2022;11:96.
253. Yu-Li Z, Chia-Li C, Shih-Jui C, Bo-Sheng W. Home-Based Intelligent Exercise System for Seniors' Healthcare: The Example of Golf Croquet. Sports. 2023;11.
254. Yuhan Z, Longxiang L, Xiuli W. Aging with robots: a brief review on eldercare automation. Interdisciplinary Nursing Research. 2024.
255. Yulei S, Jiaojiao G, Yuqing C, Jiarui S, Xueqing Z, Dan L, et al. Nursing Robots Can Reduce Nursing Workload in General Adult Wards: A Two-Phase Study. Journal of Nursing Management. 2025;2025.
256. Yunho J, Joonho M, YoungJun K. Key Performance Indicators for Service Robotics in Senior Community-Based Settings. Healthcare. 2025;13.
257. Zhao I, Angela YML, Yaqi H, Yaqian L. A Social Robot in Home Care: Acceptability and Utility Among Community-Dwelling Older Adults. Innovation in Aging. 2025;9.

Protocol

Service Robots as Work Support for Health Personnel in Long-term Care: Protocol for a Scoping Review

Diego Losada-Floriano MD, MSc; Elin Thygesen PhD; Filippo Sanfilippo PhD; Michael Rygaard Hansen PhD; Mariann Fossum PhD.

Source: Scopus AI. Current version accessible via the University of Agder Library's Scopus account, developed by Elsevier (2025). The search was conducted on December 16, 2025.

#	Prompt	Records retrieved
**1**	*"How can service robots improve the quality of healthcare services, optimise resource utilisation, and improve working conditions for healthcare personnel in long-term care settings?"*	73 references
**2**	*"In what ways can service robots enhance the quality of healthcare services, optimize resource utilization, and improve working conditions for healthcare personnel in long-term care institutions by assuming routine and repetitive tasks associated with the care of older adults?"*	10 references

Results:

1. Acun C, Ashary A, Popa DO, Nasraoui O, editors. Task Allocation for Nursing Robots Using Explainable Machine Learning with Factorization Machines. IEEE International Conference on Automation Science and Engineering; 2025.
2. Asl AM, Kouters S, Castro-González Á, Van der Roest H, Martin MF, Dröes RM. Potential Facilitators of and Barriers to Implementing the MINI Robot in Community-Based Meeting Centers for People With Dementia and Their Carers in the Netherlands and Spain: Explorative Qualitative Study. Journal of Medical Internet Research. 2023;25. doi: 10.2196/44125.
3. Athira KA, Divya Udayan J, Subramaniam U. A Systematic Literature Review on Multi-Robot Task Allocation. ACM Computing Surveys. 2024;57(3). doi: 10.1145/3700591.
4. Battistuzzi L, Papadopoulos C. The ethics of socially assistive robots in health and social care. Transcultural Artificial Intelligence and Robotics in Health and Social Care2022. p. 59–81.
5. Bersani MM, Camilli M, Lestingi L, Mirandola R, Rossi M, Scandurra P, editors. Architecting Explainable Service Robots. Lecture Notes in Computer Science (including subseries Lecture Notes in Artificial Intelligence and Lecture Notes in Bioinformatics); 2023.
6. Boch A, Ryan S, Kriebitz A, Amugongo LM, Lütge C. Beyond the Metal Flesh: Understanding the Intersection between Bio- and AI Ethics for Robotics in Healthcare. Robotics. 2023;12(4). doi: 10.3390/robotics12040110.
7. Bradwell HL, Winnington R, Thill S, Jones RB, editors. Longitudinal diary data: Six months real-world implementation of affordable companion robots for older people in supported living. ACM/IEEE International Conference on Human-Robot Interaction; 2020.
8. Chen J, Ding X, Yu F. Research on the Ethical Dilemmas of Nursing Robots Assisted Home-based Elderly Care: Based on Value Sensitive Design Theory. Chinese Medical Ethics. 2023;36(12):1350–7. doi: 10.12026/j.issn.1001-8565.2023.12.09.
9. Chen SC, Moyle W, Jones C, Petsky H. A social robot intervention on depression, loneliness, and quality of life for Taiwanese older adults in long-term care. International Psychogeriatrics. 2020;32(8):981–91. doi: 10.1017/S1041610220000459.
10. Cruz-Sandoval D, Morales-Tellez A, Sandoval EB, Favela J, editors. A social robot as therapy facilitator in interventions to deal with dementia-related behavioral symptoms. ACM/IEEE International Conference on Human-Robot Interaction; 2020.
11. Das GP, McGinnity TM, Coleman SA, Behera L. A Distributed Task Allocation Algorithm for a Multi-Robot System in Healthcare Facilities. Journal of Intelligent and Robotic Systems: Theory and Applications. 2015;80(1):33–58. doi: 10.1007/s10846-014-0154-2.
12. Esposito R, Bonaccorsi M, Fiorini L, Manzi A, Limosani R, Cavallo F, et al. Supporting active and healthy aging with advanced robotics integrated in smart environment. Artificial Intelligence: Concepts, Methodologies, Tools, and Applications2016. p. 2656–86.

13. Feng Y, Barakova EI, Yu S, Hu J, Matthias Rauterberg GW. Effects of the level of interactivity of a social robot and the response of the augmented reality display in contextual interactions of people with dementia. Sensors (Switzerland). 2020;20(13):1–12. doi: 10.3390/s20133771.
14. Fosch Villaronga E, Roig A. European regulatory framework for person carrier robots. Computer Law and Security Review. 2017;33(4):502–20. doi: 10.1016/j.clsr.2017.03.018.
15. Fosch-Villaronga E, Calleja CJ, Drukarch H, Torricelli D. How can ISO 13482:2014 account for the ethical and social considerations of robotic exoskeletons? Technology in Society. 2023;75. doi: 10.1016/j.techsoc.2023.102387.
16. Franke A, Nass E, Piereth AK, Zettl A, Heidl C. Implementation of Assistive Technologies and Robotics in Long-Term Care Facilities: A Three-Stage Assessment Based on Acceptance, Ethics, and Emotions. Frontiers in Psychology. 2021;12. doi: 10.3389/fpsyg.2021.694297.
17. Garg S, Srivastava SP, Malhotra RK, Sharma R, editors. Sustainable Integration of Robotic Services in the Hospitality Industry: Enhancing Operational Efficiency and Employee Satisfaction. 2025 International Conference on Intelligent Control, Computing and Communications, IC3 2025; 2025.
18. Gasteiger N, Ahn HS, Lee C, Lim J, MaCdonald BA, Kim GH, et al. Participatory Design, Development, and Testing of Assistive Health Robots with Older Adults: An International Four-year Project. ACM Transactions on Human-Robot Interaction. 2022;11(4). doi: 10.1145/3533726.
19. Gebellí F, Ros R. An in-situ participatory approach for assistive robots: methodology and implementation in a healthcare setting. Frontiers in Robotics and AI. 2025;12. doi: 10.3389/frobt.2025.1648737.
20. Getson C, Nejat G. Human-robot interactions with an autonomous health screening robot in long-term care settings. Advanced Robotics. 2023;37(24):1576–90. doi: 10.1080/01691864.2023.2293133.
21. Gong T. The effect of service robots on employees' customer service performance and service-oriented organizational citizenship behavior. Journal of Service Theory and Practice. 2025;35(2):319–47. doi: 10.1108/JSTP-04-2024-0104.
22. Horstmannshoff C, Jahn ET, Müller M. Integrating the Perspectives of Relevant Stakeholders into the Development of a Service Robot for Nursing Homes: Needs Analysis and Scenario Development Using the International Classification of Functioning, Disability and Health (ICF). International Journal of Social Robotics. 2025;17(9):1725–45. doi: 10.1007/s12369-024-01205-2.
23. Hsieh CJ, Li PS, Wang CH, Lin SL, Hsu TC, Tsai CMT. Socially Assistive Robots for People Living with Dementia in Long-Term Facilities: A Systematic Review and Meta-Analysis of Randomized Controlled Trials. Gerontology. 2023;69(8):1027–42. doi: 10.1159/000529849.
24. Hu R, Iturralde K, Linner T, Zhao C, Pan W, Pracucci A, et al. A simple framework for the cost-benefit analysis of single-task construction robots based on a case study of a cable-driven facade installation robot. Buildings. 2021;11(1):1–17. doi: 10.3390/buildings11010008.
25. Hung L, Gregorio M, Mann J, Wallsworth C, Horne N, Berndt A, et al. Exploring the perceptions of people with dementia about the social robot PARO in a hospital setting. Dementia. 2021;20(2):485–504. doi: 10.1177/1471301219894141.
26. Hung L, Wong JOY, Ren H, Zhao Y, Fu JJ, Mann J, et al. The Impact of Telepresence Robots on Family Caregivers and Residents in Long-Term Care. International Journal of Environmental Research and Public Health. 2025;22(5). doi: 10.3390/ijerph22050713.
27. Iglesias A, Jose RVA, Perez-Lorenzo M, Ting KLH, Tudela A, Marfil R, et al., editors. Towards long term acceptance of Socially Assistive Robots in retirement houses: Use case definition. 2020 IEEE International Conference on Autonomous Robot Systems and Competitions, ICARSC 2020; 2020.
28. Jacobs T, Graf B, editors. Practical evaluation of service robots for support and routine tasks in an elderly care facility. Proceedings of IEEE Workshop on Advanced Robotics and its Social Impacts, ARSO; 2012.
29. Jacobs T, Graf B, editors. Practical evaluation of service robots for support and routine tasks in an elderly care facility. Proceedings of IEEE Workshop on Advanced Robotics and its Social Impacts, ARSO; 2012.
30. Jenamani RK, editor. Towards Deployable Physical Caregiving Robots: A Case Study in Mealtime Assistance. ACM/IEEE International Conference on Human-Robot Interaction; 2025.
31. Ji Y, Moon J, Kim Y. Key Performance Indicators for Service Robotics in Senior Community-Based Settings. Healthcare (Switzerland). 2025;13(7). doi: 10.3390/healthcare13070770.
32. Ji Y, Moon J, Kim Y. Key Performance Indicators for Service Robotics in Senior Community-Based Settings. Healthcare (Switzerland). 2025;13(7). doi: 10.3390/healthcare13070770.

33. Johnson MJ, Johnson MA, Sefcik JS, Cacchione PZ, Mucchiani C, Lau T, et al. Task and Design Requirements for an Affordable Mobile Service Robot for Elder Care in an All-Inclusive Care for Elders Assisted-Living Setting. International Journal of Social Robotics. 2020;12(5):989–1008. doi: 10.1007/s12369-017-0436-5.
34. Jung JI, Jeong YS, Kwon DR. The effectiveness of care robots in alleviating physical burden and pain for caregivers: Non-randomized prospective interventional study - Preliminary study. Medicine (United States). 2024;103(50):e40877. doi: 10.1097/MD.0000000000040877.
35. Kato K, Yoshimi T, Aimoto K, Sato K, Itoh N, Kondo I. Reduction of multiple-caregiver assistance through the long-term use of a transfer support robot in a nursing facility. Assistive Technology. 2023;35(3):271–8. doi: 10.1080/10400435.2022.2039324.
36. Kaur A, Goyal S. Explainable AI in Healthcare: Introduction. Explainable Artificial Intelligence in the Healthcare Industry2025. p. 307–23.
37. Kim Y, Ryu H. Assistive Devices and Caregiver Retention in Long-Term Care Institutions. Research on Aging. 2025;48(5-6):312–22. doi: 10.1177/01640275251380532.
38. Kim YS, Shin HR, Yoon HJ, Ban SW, Kim YG, Jung KW, et al. Usability study of a smart transfer-assistive robot with dual arms for care workers. Disability and Rehabilitation: Assistive Technology. 2024;19(5):1864–70. doi: 10.1080/17483107.2023.2285427.

39. Klebbe R, Friese C. Exploring the Role of Robots in Inpatient Care: Caregivers´ Perspectives on the Development and Evaluation of Collaborative Robot Applications – A Qualitative Research Approach. International Journal of Social Robotics. 2025;17(9):1707–24. doi: 10.1007/s12369-025-01270-1.
40. Kodate N, Obayashi K, Maeda Y, Yu W, O'Shea D, Sakata N, et al., editors. Care professionals' experience of deploying an original non-autonomous air-purification robot in residential care homes in Ireland and Japan. HORA 2023 - 2023 5th International Congress on Human-Computer Interaction, Optimization and Robotic Applications, Proceedings; 2023.
41. Kyounga L, Ae-Ri J, Eun-A P, Seon-Mi J. Analysis of instructors' intention to use and experience of using cognitive training robots for older adults with mild cognitive impairment. Universal Access in the Information Society. 2025;24(4):2945–56. doi: 10.1007/s10209-024-01141-y.
42. Law M, Ahn HS, Broadbent E, Peri K, Kerse N, Topou E, et al. Case studies on the usability, acceptability and functionality of autonomous mobile delivery robots in real-world healthcare settings. Intelligent Service Robotics. 2021;14(3):387–98. doi: 10.1007/s11370-021-00368-5.
43. Liedo B, Van Grunsven J, Marin L. Emotional Labor and the Problem of Exploitation in Roboticized Care Practices: Enriching the Framework of Care Centred Value Sensitive Design. Science and Engineering Ethics. 2024;30(5). doi: 10.1007/s11948-024-00511-2.
44. Loveys K, Prina M, Axford C, Domènec ÒR, Weng W, Broadbent E, et al. Artificial intelligence for older people receiving long-term care: a systematic review of acceptability and effectiveness studies. The Lancet Healthy Longevity. 2022;3(4):e286–e97. doi: 10.1016/S2666-7568(22)00034-4.
45. Macalupu V, Miller E, Martin L, Caldwell G. Human–robot interactions and experiences of staff and service robots in aged care. Scientific Reports. 2025;15(1). doi: 10.1038/s41598-025-86255-w.
46. Masala GL, Giorgi I. Artificial Intelligence and Assistive Robotics in Healthcare Services: Applications in Silver Care. International Journal of Environmental Research and Public Health. 2025;22(5). doi: 10.3390/ijerph22050781.
47. Mele C, Russo-Spena T, Di Bernardo I, Gherardi S. Affect-Based Well-Being in Caring Practices with Companion Robots. Journal of Service Research. 2025. doi: 10.1177/10946705251372448.
48. Merx Q, Steins M, Odekerken G. The role of psychological comfort with service robot reminders: a dyadic field study. Journal of Services Marketing. 2024;39(10):1–14. doi: 10.1108/JSM-12-2023-0476.
49. Moyle W, Jones C, Sung B, Bramble M, O'Dwyer S, Blumenstein M, et al. What Effect Does an Animal Robot Called CuDDler Have on the Engagement and Emotional Response of Older People with Dementia? A Pilot Feasibility Study. International Journal of Social Robotics. 2016;8(1):145–56. doi: 10.1007/s12369-015-0326-7.
50. Otaka E, Sato K, Shimotori D, Ninomiya T, Sugimoto N, Nakabo Y, et al. Longitudinal changes following the introduction of socially assistive robots in nursing homes: a qualitative study with ICF framework and causal loop diagramming. BMC Geriatrics. 2024;24(1). doi: 10.1186/s12877-024-05628-4.
51. Owlia M, Kamachi M, Dutta T. Reducing lumbar spine flexion using real-time biofeedback during patient handling tasks. Work. 2020;66(1):41–51. doi: 10.3233/WOR-203149.

52. Papadopoulos I, Lazzarino R. Developing, delivering, and evaluating an online course on socially assistive robots in culturally competent and compassionate healthcare: A sequential multiphase, mixed-method study. Digital Health. 2024;10. doi: 10.1177/20552076241271792.
53. Pedersen AKB, Skinner MS, Sogstad M. Needs assessment in long-term care: expression of national principles for priority setting in service allocation. BMC Health Services Research. 2024;24(1). doi: 10.1186/s12913-024-10889-1.
54. Pekkarinen S, Hennala L, Melkas H. Towards Technology Domestication in a Care Organization? Facilitating and Hindering Factors in Care Robot Use. International Journal of Social Robotics. 2025;17(4):655–74. doi: 10.1007/s12369-025-01226-5.
55. Poonia P, Tewolde B, Surendar D, Suganya G, Muthulekshmi M, Naghapushanam M, editors. AI-Enhanced Robotics for Efficient Hospital Facility Maintenance in Cloud Environments. Proceedings of the 2025 11th International Conference on Communication and Signal Processing, ICCSP 2025; 2025.
56. Poulsen A, Burmeister OK, Greig J, Ulhaq A, Tien D. Value Sensitive Design of Social Robots: Enhancing the Lives of LGBT+ Older Adults. International Journal of Social Robotics. 2025;17(1):147–62. doi: 10.1007/s12369-024-01201-6.
57. Randall N, Šabanović S, Chang W, editors. Engaging older adults with depression as co-designers of assistive in-home robots. PervasiveHealth: Pervasive Computing Technologies for Healthcare; 2018.
58. Ren HL, Wong KLY, Soni A, Lee K, Arora S, Banco J, et al. Feasibility and Acceptability of Deploying a Collaborative Service Robot in Long-Term Care: Staff Experiences. Electronics (Switzerland). 2025;14(7). doi: 10.3390/electronics14071247.
59. Ruan YX, Che RP, Kodate N, Donnelly S, Shi Y, Liu X, et al. A Scoping Review on Long-Term Care Workers' Perceptions of Robot-Assisted Care for Older Adults in Long-Term Care Facilities. Journal of Advanced Nursing. 2025;81(12):8409–31. doi: 10.1111/jan.16853.
60. Sadak F, Qureshi R. Ensuring trust in healthcare robotics: The essential role of explainable AI. Explainable AI in Healthcare Imaging for Medical Diagnoses: Digital Revolution of Artificial Intelligence2025. p. 1–28.
61. Saplacan D, Khaksar W, Torresen J, editors. On Ethical Challenges Raised by Care Robots: A Review of the Existing Regulatory-, Theoretical-, and Research Gaps. Proceedings of IEEE Workshop on Advanced Robotics and its Social Impacts, ARSO; 2021.
62. Sawik B, Tobis S, Baum E, Suwalska A, Kropińska S, Stachnik K, et al. Robots for Elderly Care: Review, Multi-Criteria Optimization Model and Qualitative Case Study. Healthcare (Switzerland). 2023;11(9). doi: 10.3390/healthcare11091286.
63. Shi J, Yang Z, Zhu J, editors. An Auction-Based Task Allocation Algorithm in Heterogeneous Multi-Robot System. EAI/Springer Innovations in Communication and Computing; 2020.
64. Siebelink NM, Gaasterland A, Gielissen M, van der Weegen S, Boon B, van der Poel A. Barriers and facilitators influencing implementation of care technology for people with intellectual disabilities: A cross-sectional study among care professionals. Journal of Applied Research in Intellectual Disabilities. 2024;37(5). doi: 10.1111/jar.13262.
65. Sivakanthan S, Blaauw E, Greenhalgh M, Koontz AM, Vegter R, Cooper RA. Person transfer assist systems: a literature review. Disability and Rehabilitation: Assistive Technology. 2021;16(3):270–9. doi: 10.1080/17483107.2019.1673833.
66. Sorell T, Draper H. Robot carers, ethics, and older people. Ethics and Information Technology. 2014;16(3):183–95. doi: 10.1007/s10676-014-9344-7.
67. Stegner L, Senft E, Mutlu B, editors. Situated Participatory Design: A Method for In Situ Design of Robotic Interaction with Older Adults. Conference on Human Factors in Computing Systems - Proceedings; 2023.
68. Sun J, Zhao D, Ren Z, Yang Z, Liu H, Yang J, editors. Multi-robot Task Allocation for Nursing Home Based on Improved Particle Swarm Algorithm. ICMLCA 2021 - 2nd International Conference on Machine Learning and Computer Application; 2021.
69. Takanokura M, Kurashima R, Ohhira T, Kawahara Y, Ogiya M. Implementation and user acceptance of social service robot for an elderly care program in a daycare facility. Journal of Ambient Intelligence and Humanized Computing. 2023;14(11):14423–32. doi: 10.1007/s12652-020-02871-6.
70. Takanokura M, Kurashima R, Ohhira T, Kawahara Y, Ogiya M. Implementation and user acceptance of social service robot for an elderly care program in a daycare facility. Journal of Ambient Intelligence and Humanized Computing. 2023;14(11):14423–32. doi: 10.1007/s12652-020-02871-6.

71. Tang Y, Dou B. Cost-effectiveness analysis of robotic surgery in healthcare for older individuals: a systematic review based on randomized controlled trials. Frontiers in Public Health. 2025;13. doi: 10.3389/fpubh.2025.1614654.
72. Thiuthipsakul P, Suthakorn J. Design and implementation of standardized mobile robots for optimizing medical supply distribution and patient care: Intelligent automation in healthcare. Smart Healthcare, Clinical Diagnostics, and Bioprinting Solutions for Modern Medicine2025. p. 85–99.
73. Tiberio L, Mitzner TL, Kemp CC, Rogers WA, editors. Investigating Healthcare Providers' Acceptance of Personal Robots for Assisting with Daily Caregiving Tasks. Conference on Human Factors in Computing Systems - Proceedings; 2013.
74. Tobis S, Piasek J, Cylkowska-Nowak M, Suwalska A. Robots in Eldercare: How Does a Real-World Interaction with the Machine Influence the Perceptions of Older People? Sensors. 2022;22(5). doi: 10.3390/s22051717.
75. Tu Y, Liu W, Yang Z. Exploring the influence of service employees' characteristics on their willingness to work with service robots. Journal of Service Management. 2023;34(5):1038–63. doi: 10.1108/JOSM-05-2022-0174.
76. Turja T. Uncertainties about accepting care robots. Frontiers in Digital Health. 2023;5. doi: 10.3389/fdgth.2023.1092974.
77. Turja T, Taipale S, Niemelä M, Oinas T. Positive Turn in Elder-Care Workers' Views Toward Telecare Robots. International Journal of Social Robotics. 2022;14(4):931–44. doi: 10.1007/s12369-021-00841-2.
78. Uddin Z. Applied Machine Learning for Assisted Living2022. 1–131 p.
79. Wang YF. Applying gerontechnology to dementia care: experiences from long-term care workers in Taiwan. Disability and Rehabilitation: Assistive Technology. 2026;21(1):229–40. doi: 10.1080/17483107.2025.2553842.
80. Wong KLY, Hung L, Wong J, Park J, Alfares H, Zhao Y, et al. Adoption of Artificial Intelligence–Enabled Robots in Long-Term Care Homes by Health Care Providers: Scoping Review. JMIR Aging. 2024;7. doi: 10.2196/55257.
81. Yang ACH, Choe EY, Zhang F. Pathways linking mobile service robots and quality of life of people with dementia: a literature review. Industrial Management and Data Systems. 2025;125(8):2536–63. doi: 10.1108/IMDS-05-2024-0464.
82. Ziltener A, Frei J, Lohan KS, Ospelt T, Wüst M. Service robot for laundry transport and drink distribution in nursing homes. Gerontechnology. 2024;23:1–. doi: 10.4017/GT.2024.23.S.1058.OPP.
83. Zuurbier NP, Smaling HJA. Healthcare professionals' experiences with the SARA robot in long-term care for people with dementia and people with intellectual disabilities. Digital Health. 2025;11. doi: 10.1177/20552076251375530.

Additional references identified using AI tools included in the screening process

1. Asgharian P, Panchea AM, Ferland F. A Review on the Use of Mobile Service Robots in Elderly Care. Robotics. 2022;11(6):127. PMID: doi:10.3390/robotics11060127.
2. El-Gazar HE, Abdelhafez S, Ali AM, Shawer M, Alharbi TAF, Zoromba MA. Are nurses and patients willing to work with service robots in healthcare? A mixed-methods study. BMC Nursing. 2024 2024/10/07;23(1):718. doi: 10.1186/s12912-024-02336-7.
3. Johnson MJ, Johnson MA, Sefcik JS, Cacchione PZ, Mucchiani C, Lau T, et al. Task and Design Requirements for an Affordable Mobile Service Robot for Elder Care in an All-Inclusive Care for Elders Assisted-Living Setting. International Journal of Social Robotics. 2020 2020/11/01;12(5):989–1008. doi: 10.1007/s12369-017-0436-5.
4. Jung JI, Jeong YS, Kwon DR. The effectiveness of care robots in alleviating physical burden and pain for caregivers: Non-randomized prospective interventional study – Preliminary study. Medicine. 2024;103(50):e40877. PMID: 00005792-202412130-00083. doi: 10.1097/md.0000000000040877.
5. Lee SH, Kim JS, Yu S. The impact of care robots on older adults: A systematic review. Geriatric Nursing. 2025 2025/09/01/;65:103507. doi: https://doi.org/10.1016/j.gerinurse.2025.103507.
6. Masala GL, Giorgi I. Artificial Intelligence and Assistive Robotics in Healthcare Services: Applications in Silver Care. International Journal of Environmental Research and Public Health. 2025;22(5):781. PMID: doi:10.3390/ijerph22050781.

7. Persson M, Redmalm D, Iversen C. Caregivers' use of robots and their effect on work environment – a scoping review. Journal of Technology in Human Services. 2022 2022/07/03;40(3):251–77. doi: 10.1080/15228835.2021.2000554.
8. Stegner L, Mutlu B. Designing for Caregiving: Integrating Robotic Assistance in Senior Living Communities. Proceedings of the 2022 ACM Designing Interactive Systems Conference. 2022.
9. Yoshimi T, Kato K, Tsuchimoto S, Mizuguchi N, Aimoto K, Itoh N, et al. Investigating proficiency using a lift-type transfer support device for effective care: comparison of skilled and unskilled nursing homes. Disability and Rehabilitation: Assistive Technology. 2024 2024/04/02;19(3):841–50. doi: 10.1080/17483107.2022.2128444.
