# Supplementary material for: Service Robots as Work Support for Health Personnel in Long-Term Care: Protocol for a Scoping Review
Source: JMIR Res Protoc. 2026 Jul 8;15:e89435. doi: 10.2196/89435 (PMC13392531; doi:10.2196/89435)
Supplement: Multimedia Appendix 4 [file resprot_v15i1e89435_app4.pdf]

Protocol

Service Robots as Work Support for Health Personnel in Long-term Care: Protocol for a Scoping Review

Diego Losada-Floriano MD, MSc; Elin Thygesen PhD; Filippo Sanfilippo PhD; Michael Rygaard Hansen PhD; Mariann Fossum PhD.

Multimedia Appendix 3. Data extraction instrument

Reviewer:	Reviewers' response:
Date of data extraction	
Author(s)	
Year of publication	
Country (Origin)	
Citation details (e.g. author/s, date, title, journal, volume, issue, pages)	
Methods:<br>- Qualitative<br>- Quantitative<br>- Mixed Methods<br>- Multi-methods<br>- Other (Which one?)	
Type of Publication or Evidence Source:<br>- Systematic Review<br>- Meta-analysis<br>- Randomised Controlled Trial (RCT)<br>- Non-randomised Controlled Trial<br>- Quasi-experimental Study<br>- Before-and-After Study<br>- Prospective Cohort Study<br>- Retrospective Cohort Study<br>- Case-Control Study)<br>- Cross-sectional Study<br>- Other quantitative study (Which one?)<br>- Qualitative Study<br>- Mixed Methods Study<br>- Gray Literature (Which one?)<br>- Policy Document<br>- Government Report<br>- Clinical Practice Guideline<br>- Thesis/Dissertation<br>- Institutional and Research Centre Report<br>- Health Technology Assessment (HTA)<br>- Website Content<br>- Other (Which one?)	
Aim or purpose	
Types of Participants (Target Population):	

- Health Personnel<br>- Patients (Aged)<br>- Informal caregivers or patient's family.	
Participants (Population) details or characteristics: e.g. age/sex and number (sample size).	
Concept: Definition of service robot	
Concept: Classification and/or description of technologies used in service robots	
Context: Type of Long-Term<br>Care Facility<br>- Nursing homes<br>- Assisted living facilities<br>- Residential or home care institutions/facilities	
Context: Long-term care settings, locations and/or services with service robot implementation (e.g. medical services, nursing services, physiotherapy services, social and leisure activities, etc.)	
Context: Detailed account of the applications and intervention descriptions of service robots.	
Outcomes measured and reported	
Theoretical frameworks or models used to guide the implementation	
Key findings that relate to the scoping review question (Evidence established or authors conclusions)	
Knowledge gaps reported	
